# Attributes in stated preference elicitation studies on colorectal cancer screening and their relative importance for decision-making among screenees: a systematic review

**DOI:** 10.1186/s13561-022-00394-8

**Published:** 2022-09-22

**Authors:** Melanie Brinkmann, Lara Marleen Fricke, Leonie Diedrich, Bernt-Peter Robra, Christian Krauth, Maren Dreier

**Affiliations:** 1 Institute for Epidemiology, Social Medicine and Health Systems Research, Hannover Medical School, Hannover, Germany; 2 Institute of Social Medicine and Health Systems Research, Otto-von-Guericke University Magdeburg, Magdeburg, Germany

**Keywords:** Colorectal cancer screening, Systematic review, Discrete choice experiment, Risk of bias, GRADE, Informed decision-making

## Abstract

**Introduction:**

The SIGMO study (Sigmoidoscopy as an evidence-based colorectal cancer screening test – a possible option?) examines screening eligible populations’ preferences for colorectal cancer (CRC) screening in Germany using a discrete choice experiment (DCE). Attribute identification and selection are essential for the construction of choice tasks and should be evidence-based. As a part of the SIGMO study this systematic review provides an overview of attributes included in studies eliciting stated preferences for CRC screening tests and their relative importance for decision-making.

**Methods:**

Systematic search (November 2021) for English-language studies published since January 2000 in PubMed, Embase, Web of Science, Biomedical Reference Collection: Corporate Edition, LIVIVO and PsycINFO. DCEs and conjoint analysis ranking or rating tasks on screening eligible populations’ preferences for stool testing, sigmoidoscopy, and/or colonoscopy were included. Attributes were extracted and their relative importance was calculated and ranked. Risk of bias (RoB) of included studies was assessed using a modified GRADE (Grading of Recommendations Assessment, Development and Evaluation) approach. Study selection and RoB rating were carried out independently by two reviewers. Data were extracted by one reviewer and checked by another one.

**Results:**

A total of 23 publications on 22 studies were included. Overall RoB was rated as serious/critical for 21 studies and as moderate for 2 studies. Main reasons for high RoB were non-random sampling, low response rates, lack of non-responder analyses, and, to a lesser extent, weaknesses in the measurement instrument and data analysis. Extracted attributes (*n* = 120) referred to procedure-related characteristics (*n* = 42; 35%), structural characteristics of health care (*n* = 24; 20%), test characteristics (*n* = 23; 19%), harms (*n* = 16; 13%), benefits (*n* = 13; 11%), and level of evidence (*n* = 2; 2%). Most important attributes were reduction in CRC mortality (and incidence) (*n* = 7), test sensitivity (*n* = 7), out-of-pocket costs (*n* = 4), procedure (*n* = 3), and frequency (*n* = 2).

**Conclusions:**

Health preference studies on CRC were found to have a high RoB. The composition of choice tasks revealed a lack of attributes on patient-important outcomes (like incidence reduction), while attributes not considered relevant for individual screening decisions (like sensitivity) were frequently used. Future studies eliciting stated preferences in cancer screening should apply the principles of informed decision-making in attribute identification and selection.

**Supplementary Information:**

The online version contains supplementary material available at 10.1186/s13561-022-00394-8.

## Introduction

Limited health care resources require prioritisation or rationing of health services and goods [1–3]. Regulatory decisions on health care (e.g., reimbursement decisions) should take into account the needs and preferences of patients and the public as potential beneficiaries [3, 4]. The U.S. Food and Drug Administration (FDA) [5] notes that considering patient preferences is particularly important in preference-sensitive decisions where multiple treatment options are available but none is clearly superior to the others for all patients. To elicit preferences in the context of the benefit-risk assessment of health services or goods, the FDA [5] and the German Institute for Quality and Efficiency in Health Care [6] recommend, among others, stated preference methods such as the choice-based conjoint analysis (CA), also known as discrete choice experiment (DCE).

A DCE is a multi-attribute preference elicitation method [7]. The respondents are usually presented with several choice tasks (choice sets), each comparing two or more (hypothetical) alternatives. In each choice set, respondents are asked to choose the alternative they most prefer. The alternatives are defined by several attributes (e.g., frequency of screening test) with different levels (e.g., every year – every 5 years – every 10 years) assigned to each of them [2, 7, 8]. Based on the choices made, the relative importance of the attributes, trade-offs between them and the predicted uptake of health services or products can be determined [4, 9].

Within preference elicitation research, the choice of colorectal cancer (CRC) screening is becoming increasingly recognized (e.g., [10, 11]). CRC was the third most commonly diagnosed cancer and the second leading cause of cancer deaths worldwide in 2020 [12]. Recommendations for CRC screening differ between countries. The most frequently recommended screening methods are, however, faecal occult blood testing (FOBT), either guaiac-based (gFOBT) or more recently immunochemical (FIT), flexible sigmoidoscopy and colonoscopy [13–17]. In Germany, everyone who is eligible for screening within the statutory health insurance according to age can decide between FIT and colonoscopy as part of an organised, quality-assured screening programme [18]. Although sigmoidoscopy, proven to reduce CRC incidence and mortality, is recommended for individuals rejecting the screening colonoscopy, it is not covered by statutory health insurance [18, 19].

The SIGMO study (Sigmoidoscopy as an evidence-based colorectal cancer screening test – a possible option?) analyses screening eligible populations’ preferences for CRC screening in Germany using a DCE [20]. To construct preference elicitation tasks, the identification and selection of attributes are an essential step and should be supported by evidence [7]. Systematic reviews of studies eliciting average-risk populations’ preferences for cancer screening in general [21–23] or CRC screening [10, 11, 24] have already been conducted. However, the most recent review on CRC screening specific attributes covered a search period up to April 2013 [11]. Furthermore, an assessment of the risk of bias of the included studies is lacking in previous systematic reviews [23, 25, 26]. To enable an evidence-based attribute identification and selection for the DCE conducted in the SIGMO study, as well as for future stated preference elicitation studies related to CRC screening, the objectives of this systematic review were to 1) provide an overview of the attributes that have been included in CAs or DCEs eliciting screening eligible populations’ preferences for CRC screening tests, and 2) to analyse the relative importance of attributes for informed decision-making.

## Methods

The conduct and reporting of this systematic review was based on the Preferred Reporting Items for Systematic reviews and Meta-Analyses (PRISMA) Statement [26] (see Additional file 1 for PRISMA checklist). There is no separate review protocol, as this systematic review was conducted as a part of the SIGMO study. The SIGMO study is registered at the German Clinical Trials Register (DRKS00019010), a study protocol was published [20].

### Eligibility criteria

Eligible for inclusion were DCEs and CA ranking or rating tasks on preferences of the screening eligible population for at least one of the following CRC screening tests: FOBT (gFOBT or FIT), sigmoidoscopy and/or colonoscopy. Only primary research and English-language studies published since January 2000 were considered. The date restriction was applied due to actuality reasons. In addition, it was not until the early 1990s that DCEs were implemented in health economics [8]. The period from 1990 to 2000 was covered by earlier systematic reviews on stated preferences for (colorectal) cancer screening [11, 21, 22, 24] but without finally including a study published before 2000.

### Search strategy

Studies were identified by systematic search in the bibliographic databases PubMed, Embase, Web of Science, Biomedical Reference Collection: Corporate Edition, LIVIVO and PsycINFO. Additionally, reference lists of included studies were screened. In accordance with our eligibility criteria, the date range covered for each of the electronic databases was from 2000 to present. No other limits were applied. The last search was run on 18 November 2021.

Our search strategy combined database specific controlled vocabulary search terms with a wide range of free-text terms including spelling variants, synonyms and truncation [25] related to the following categories: screening test, colorectal cancer, screening, colorectal cancer screening, and preferences. See Additional file 2 for the search terms used and the full electronic search strategy applied to each database.

### Study selection

Study selection was performed independently by two reviewers (MB and LMF/DS) by initially screening titles and abstracts, followed by full text screening for compliance with our eligibility criteria. Disagreements on whether or not a record met our eligibility criteria were resolved by consensus between the reviewers, and, if necessary, including a third person (MD).

### Data collection process

We developed a data extraction form that was piloted and further refined. One reviewer (MB) extracted the data from the included studies. A second reviewer (KT) checked the extracted data; changes were made based on a discussion between the two reviewers.

Data were extracted on 1) general information (authors, title, year and journal of publication, objective, country, study duration, preference elicitation method, funding, included screening tests), 2) attributes and levels, and 3) utility values (preference weights, importance values).

We combined the extracted attributes in the categories procedure-related characteristics, test characteristics, benefits, harms, structural characteristics of health care, and level of evidence. The assignment as well as the naming of these categories were achieved inductively in an iterative consensus process considering literature regarding the recommended contents of evidence-based health information and decision aids [27–29]. Test characteristics include sensitivity, specificity, 1-sensitivity, 1-specificity [30, 31], and the proportion of false positive test results in relation to all screened persons. The latter comprises attributes that, for example, have been referred to as the number of unnecessary colonoscopies caused by the possible occurrence of false-positive results per overall count of people who took part in screening. Due to heterogeneity in the naming of attributes that could be assigned to test characteristics, the final classification was based on 1) attribute descriptions as given by the authors of included studies, 2) frequencies presented, which were summarised in the corresponding cells of a two-by-two table, 3) checking corresponding levels for plausibility, and 4) consenting in our research team. Out-of-pocket costs were attributed to the structural characteristics of health care and not to harms in a broader sense, as it depends on the health care system whether individuals incur costs for CRC screening offers or not.

### Calculating the relative importance of attributes

Attributes with at least one attribute-level preference weight (β coefficient) reported as being significant at *p* ≤ 0.05 by the authors of included studies were considered in the analysis of relative importance. In studies, where various models were estimated, only preference weights from main effects models, models providing the best fit, or models based on the total sample rather than subgroups were included. Attribute-level preference weights were extracted and relative importance within each study was calculated as follows: 1) generating attribute utility ranges between the highest and lowest β coefficient of attribute specific levels, 2) summing up all attribute utility ranges, 3) dividing the individual attribute utility ranges by the sum total of attribute utility range, 4) determining the relative importance (in %), and 5) providing an importance ranking of the attributes within a study [9, 32, 33]. For studies that reported attribute-level preference weights for subgroups only, more than one relative importance ranking was calculated. Attributes with the highest relative importance values were scored as most important. The coefficients of continuous attributes were multiplied by the range of the related levels when the measurement unit of the respective coefficient was specified. If the reference value was not made explicit, the authors were contacted via Email. In cases where no clarifying response was received, 1) the measurement unit was estimated if there were any indications found in the publication, 2) the relative importance was adopted as reported by the authors, or 3) the respective study was excluded from analysis. In studies where an importance score was given, these frequencies were extracted as a measure of relative importance.

### Risk of bias in individual studies

Risk of bias in included studies was assessed using the approach addressing the certainty of evidence in the relative importance of outcomes or values and preferences developed by The Grading of Recommendations Assessment, Development and Evaluation (GRADE) working group [34]. We used the risk of bias domain with the following four subdomains: selection of participants into the study, completeness of data, measurement instrument and data analysis. Overall risk of bias of a study was rated as low, moderate, serious or critical, and was assigned corresponding to the highest risk of bias identified in at least one subdomain. The approach was developed to be applied to a wide range of different measurements of the relative importance of outcomes. We thus adapted the subdomains, particularly measurement instrument and data analysis, to the requirements for a low risk of bias rating to methodological quality standards of DCEs and CAs (Additional file 3) by taking into account literature on good research practice for these preference elicitation methods [2, 7–9, 35].

Two reviewers (MB and LMF) completed the risk of bias rating for each of the included studies independently. Disagreements regarding the final judgement of the risk of bias within the individual studies were resolved by consensus. Where no agreement could be reached, the opinion of the study team was sought.

## Results

### Study selection

**Fig. 1:**
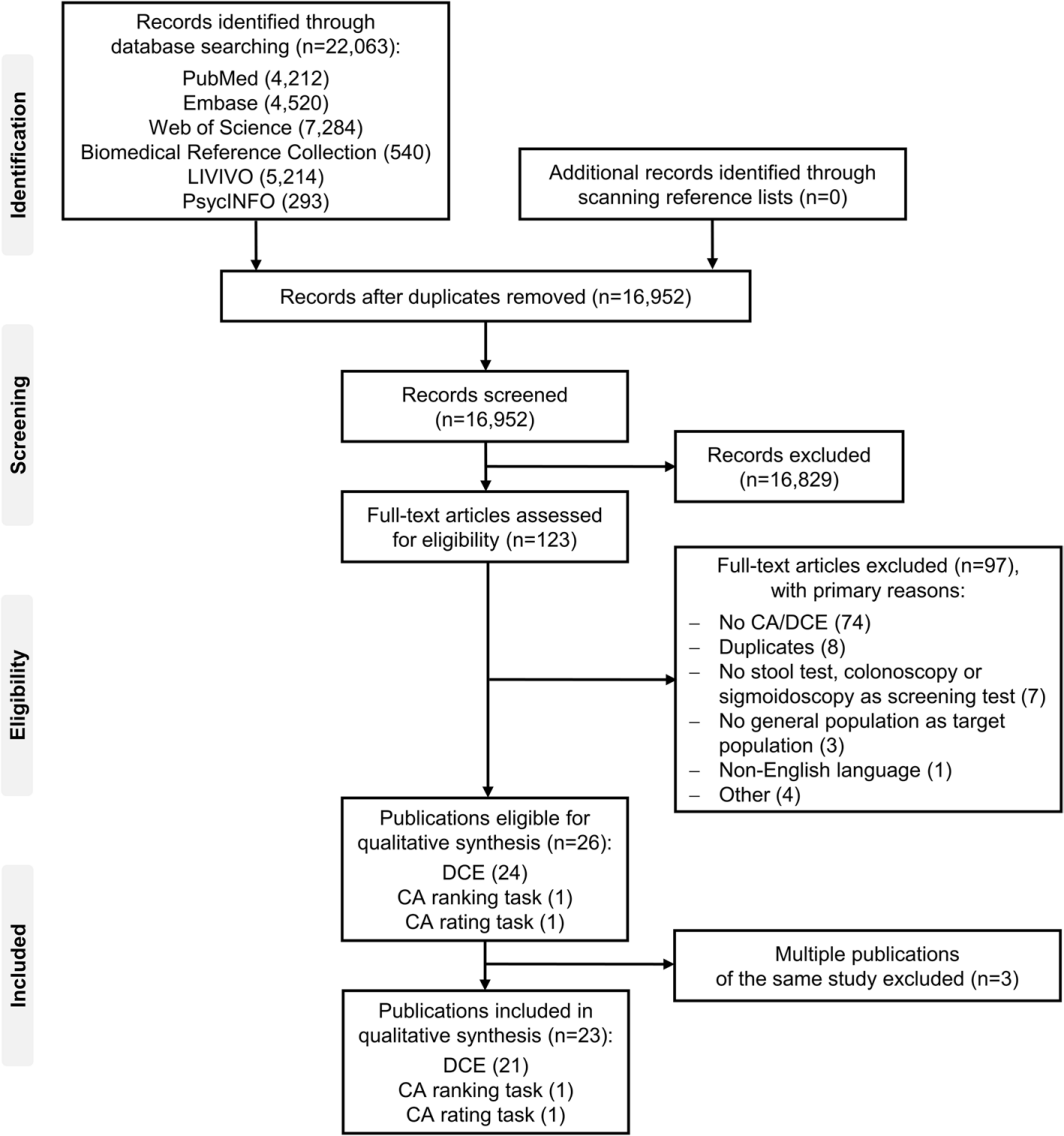
Flow diagram of study selection. Legend: *CA* conjoint analysis, *DCE* discrete choice experiment

Of 22,063 records initially identified, 23 publications on 22 studies were included in the qualitative synthesis of our systematic review (see flow diagram in Fig. 1 and Additional file 4 for an overview of the excluded records due to full-text screening with primary reasons for exclusion).

Among the 26 publications considered eligible for inclusion, multiple reports of three studies were identified. First, van Dam et al. [36], De Bekker-Grob et al. [37], and Hol et al. [38] each published different aspects out of one study. Van Dam et al. [36] and Hol et al. [38] separately report the results for a generic and labelled DCE, respectively, whereas the publication by De Bekker-Grob et al. [37] focuses on a methodological investigation of differences between these two ways of conceptualizing a choice process. Second, the publications of Marshall et al. [39] and Cheng et al. [40] also refer to one study. Marshall et al. [39] published the results of the DCE, while the article of Cheng et al. [40] focuses on a methodological exploration of different statistical models for analysing DCE data. Third, the two publications by De Bekker-Grob et al. [41] and De Bekker-Grob et al. [42] report results from one study, but focus on different content and methodological issues. For our qualitative analysis, only the publications of van Dam et al. [36], Hol et al. [38], Marshall et al. [39], and De Bekker-Grob et al. [41] were considered. The publications by De Bekker-Grob et al. [37], Cheng et al. [40], and De Bekker-Grob et al. [42] were excluded but used to complete information where necessary.

Four studies [41, 43–45] focused on the exploration of methodological issues related to the collection and analysis of preference data. Nevertheless, these studies were included because they contain relevant information regarding the objectives of this systematic review.

### Study characteristics

See Table 1 for an overview of the characteristics of included studies. All records were published between 2000 and 2021. Most studies were conducted in the USA (*n* = 8, [44–51]), the Netherlands (*n* = 6, [36, 38, 41, 52–54]), and Australia (*n* = 5, [43, 45, 55–57]). Two studies were realised in Canada [39, 47] and 1 each in Denmark [58], France [59], Great Britain [53], Iran [60], and Thailand [61]. The studies by Marshall et al. [47], Brenner et al. [45] and Groothuis-Oudshoorn et al. [53] were carried out in two countries each. The preference elicitation method most frequently used was a DCE (*n* = 21), whereas Hawley et al. [46] and Gyrd-Hansen et al. [58] applied a CA rating and ranking task, respectively. Preference elicitation tasks were labelled by Hol et al. [38], Benning et al. [52] and Benning et al. [54] and were generic in all other studies. The number of attributes included in the studies ranged from 2 (*n* = 1, [38]) to 9 (*n* = 2, [47, 60]) with most studies (*n* = 11) defining their preference elicitation tasks based on 4 to 5 attributes [41, 45, 46, 48–52, 54, 55, 58].

**Table 1:** Characteristics of included studies (*n* = 23)

Study, year, country	Objective(s)	CRC screening tests	No. of attributes	Procedure-related characteristics	Test characteristics	Benefits	Harms	Structural characteristics of health care	Level of evidence	Risk of bias^a^
**Rating**										
Hawley et al., 2008, USA [46]	To analyse preferences for CRC screening tests of racially/ethnically diverse primary care patients	FOBT, SIG, COL, DCBE, FIT, V-COL	5	✓	✓		✓			Critical
**Ranking**										
Gyrd-Hansen et al., 2001, Denmark [58]	To analyse public preferences for attributes associated with participation in cancer screening programmes	FOBT	4	✓	✓	✓		✓		Serious
**Discrete choice**										
**Generic**										
Salkeld et al., 2000, Australia [55]	To measure consumer preferences for an existing and a hypothetical new CRC screening test	Bowel scan test kit (status quo) and a hypothetical new bowel test	5	✓	✓			✓		Critical
Salkeld et al., 2003, Australia [56]	To elicit community preferences for CRC screening by faecal occult blood test based on harms and benefits	FOBT	3		✓	✓		✓		Serious
Marshall et al., 2007, Canada [39]	To analyse preferences for various CRC screening tests	FOBT, SIG, COL, DCBE, DNA stool tests, V-COL	6	✓	✓		✓	✓		Moderate
Howard et al., 2009, Australia [43]	To explore the effect of attribute framing within the context of CRC screening preferences.^b^	FITs	6	✓	✓			✓		Moderate
Marshall et al., 2009, Canada, USA [47]	To analyse and compare general-population and physician preferences for attributes of CRC screening tests	FOBT, SIG, COL, DCBE, DNA stool tests, V-COL	9	✓	✓		✓	✓		Critical
Van Dam et al., 2010, The Netherlands [36]	To analyse how procedural characteristics of CRC screening tests determine preferences for participation and how individuals weigh these against the expected health benefits from participating in CRC screening	FOBT, SIG, COL	7	✓		✓	✓			Critical
Nayaradou et al., 2010, France [59]	To identify population preferences for CRC screening test characteristics	Stool test, blood test	7	✓	✓	✓		✓		Serious
Pignone et al., 2012, USA [44]	To compare two methods for eliciting and clarifying patient values for decision-making aboutCRC screening.^b^	FOBT, SIG, COL, CT-COL	6	✓		✓	✓	✓		Critical
Brenner et al., 2014, USA, Australia [45]	To compare the effects of three methods of values clarification on decision-making about CRC screening.^b^	FOBT, SIG, COL, radiological testing	5	✓		✓	✓			Critical
Groothuis-Oudshoorn et al., 2014, The Nerlands, UK [53]	To analyse public preferences for various CRC screening tests	FIT, SIG, COL, nanopill	6	✓	✓		✓			Serious
Pignone et al., 2014, USA [48]	To analyse how vulnerable populations value different aspects of CRC screening tests	Stool test, COL, CT-COL	4	✓				✓		Critical
Kistler et al., 2015, USA [49]	To analyse older adults’ preferences for CRC screening tests	FOBT, SIG, COL	4	✓		✓	✓			Critical
Martens et al., 2016, USA [50]	To analyse preferences of the Hispanic immigrant community in North Carolina for CRC screening test characteristics and barriers and facilitators around CRC screening	Stool test, COL, CT COL	4	✓				✓		Critical
Osborne et al., 2018, Australia [57]	To analyse population preferences for CRC screening tests	Stool test, blood test, saliva test	3	✓	✓			✓		Serious
Mansfield et al., 2018, USA [51]	To analyse preferences for the features of CRC screening tests	FOBT, FIT, SIG, COL	5	✓	✓		✓	✓		Critical
Ramezani_Doroh et al., 2019, Iran [60]	To analyse the preferences for CRC screening tests	gFOBT, FIT, SIG, COL, DCBE, stool DNA test	9	✓	✓	✓	✓	✓		Critical
De Bekker-Grob et al., 2019, The Netherlands [41]	To determine whether the number of alternatives in a DCE choice task should reflect the actual decision context, and how complex the choice model needs to be to be able to predict real-world healthcare choices.^b^	FOBT	5	✓	✓	✓		✓		Serious
Phisalprapa et al., 2021, Thailand [61]	To analyse preferences and willingness to pay of individuals at risk of CRC	FIT, SIG, COL, DCBE, CT COL	6	✓		✓	✓	✓		Critical
**Labelled**										
Hol et al., 2010, The Netherlands [38]	To analyse preferences for and to predict the uptake of CRC screening tests	FOBT, SIG, COL	2	✓		✓				Critical
Benning et al., 2014, The Netherlands [52]	To analyse potential screening participants’ preferences for different non-invasive CRC screening tests	Stool test, blood test, combi test	4		✓	✓			✓	Critical
Benning et al., 2014, The Netherlands [54]	To analyse how much individuals’ participation decision in non-invasive screening is affected by the presence or absence of detailed information about invasive follow-up testing	Stool test, blood test, combi test	5		✓	✓		✓	✓	Critical

*COL* Colonoscopy, *CRC* Colorectal cancer, *CT-COL* Computed tomographic colonography, *DCBE* Double-contrast barium enema, *DCE* Discrete choice experiment, *DNA* Deoxyribo nucleic acid, *FIT* Faecal immunochemical test, *FOBT* Faecal occult blood testing, *gFOBT* guaiac-based FOBT, *SIG* (flexible) Sigmoidoscopy, *V-COL* Virtual colonoscopy

^a^Judgement of overall risk of bias within a study include low, moderate, serious or critical

^b^Exploring methodological issues

All studies included at least one stool-based test (gFOBT and/or FIT) with 4 studies [41, 43, 56, 58] eliciting preferences for different stool tests only. Twelve studies [36, 38, 39, 44–47, 49, 51, 53, 60, 61] considered both sigmoidoscopy and colonoscopy, while 2 studies [48, 50] included only colonoscopy as an endoscopic screening test. Radiologic CRC screening methods (computed tomographic colonography or virtual colonoscopy and double contrast barium enema) were taken into account in 9 studies [39, 44–48, 50, 60, 61], accompanied by at least one endoscopic procedure and one stool test. Three studies [39, 47, 60] analysed preferences for genetic stool tests, 4 studies [52, 54, 57, 59] included blood and saliva tests, and 1 study [53] assessed preferences for capsule endoscopy.

### Risk of bias within studies

**Fig. 2:**
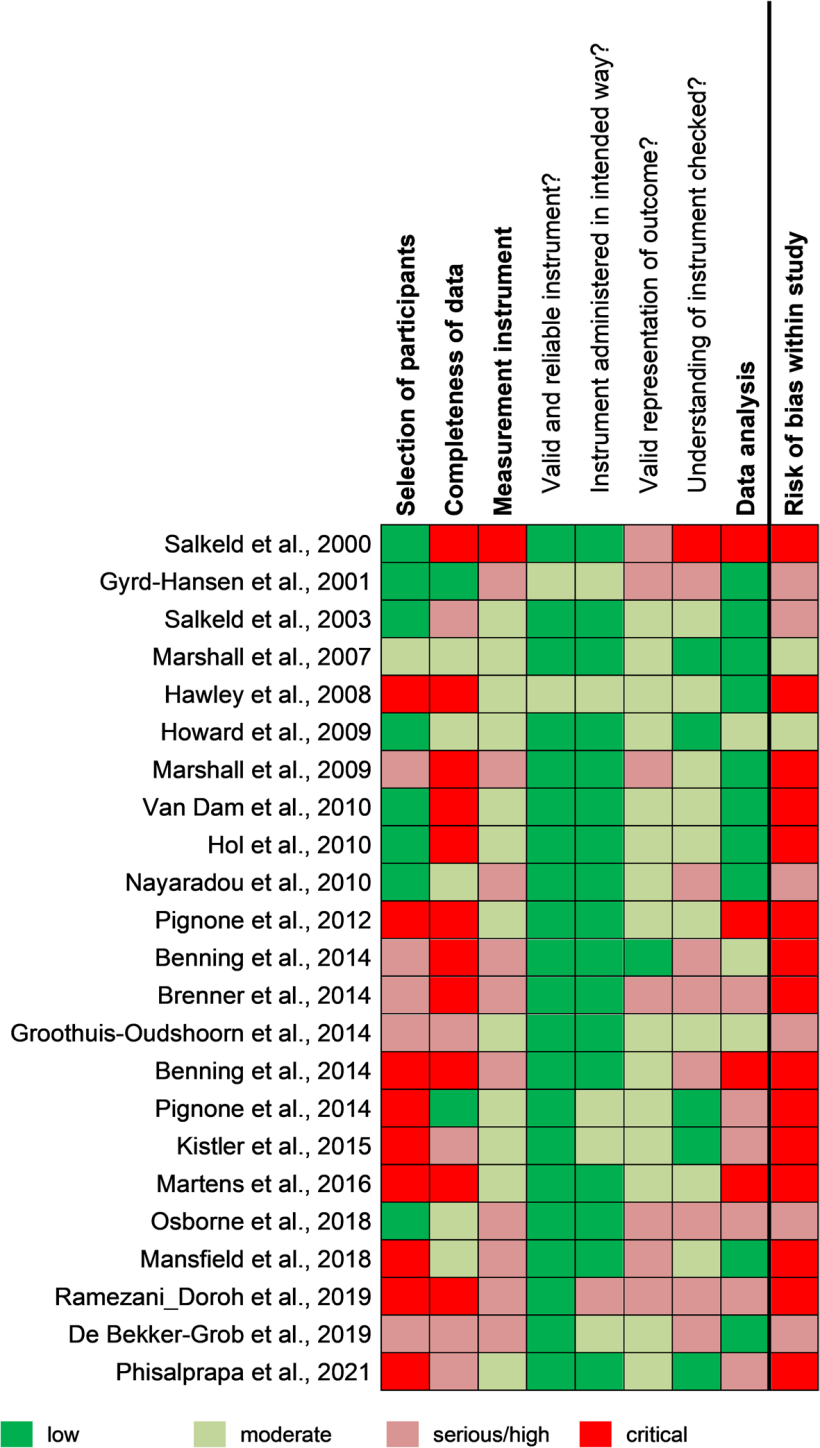
Risk of bias rating (*n* = 23)

We rated overall risk of bias as serious or critical for 21 studies and as moderate for 2 studies [39, 43] (Table 1, Fig. 2, and Additional file 5 for consensus answers and ratings including free-text support and direct quotations for each study). A higher risk of bias was more often present in the subdomains selection of participants and completeness of data than in measurement instrument and data analysis, the last two specifically addressing methodological aspects of DCEs.

A weakness of several studies in selecting participants was a non-random sampling. Instead, individuals were recruited through non-probability sampling methods like opt-in panels (e.g., [41, 45, 47, 51–54]) or convenience and purposeful (e.g., [46, 48, 50, 61]) approaches prone to selection bias. Thus, 14 studies [41, 44–54, 60, 61] were rated with serious or critical risk of bias for this subdomain. Completeness of data was classified as serious or critical risk of bias in 16 studies because response rates were low and differences between the characteristics of participants who responded and those who did not were not examined [36, 38, 41, 44–47, 49, 50, 52–56, 60, 61]. The requirements for a valid presentation of the outcome including an evidence-based and justified (e.g., literature reviews, qualitative research, and expert discussions) identification and selection of the attributes and their levels as well as sufficient explanation of the choice tasks, were met by 1 study [52]. All other studies implemented at least one of these aspects. Moreover, 21 [36, 38, 39, 41, 43–56, 59–61] studies conducted an evidence-based attribute identification and a justified attribute selection. Checking the understanding of preference elicitation tasks involves face-to-face pretest interviews and internal validity tests. Five studies [39, 43, 48, 49, 61] met both criteria. One study [55] did not test understanding at all. The subdomain data analysis addressed whether preference heterogeneity among individuals was adequately accounted for by stratification and/or interactions between socioeconomic characteristics and attributes and/or latent class models. Ten studies [36, 38, 39, 41, 46, 47, 51, 56, 58, 59] qualify with a low risk of bias in this subdomain, while 4 studies [44, 50, 54, 55] did not meet any of the criteria and were rated with critical risk of bias.

### Results of individual studies

A total of 120 attributes were extracted (Table 2). Attributes on procedure-related characteristics were used most frequently (*n* = 42; 35%), followed by 24 (20%) attributes on structural characteristics of health care and 23 (19%) attributes addressing test characteristics. Harms (*n* = 16; 13%) and benefits (*n* = 13; 11%) were used less frequently, the level of evidence was included two times (2%).

**Table 2:** Attributes (*n* = 120) of colorectal cancer screening tests by categories (*n* = 6) extracted from 23 publications

Categories	Procedure-related characteristics (*n* = 42)								Test characteristics (*n* = 23)				
Attributes	Procedure	Frequency	Preparation	Location	Follow-up test required	Duration	Mode of test delivery	Purpose	Sensitivity	Specificity	1-Speificity	1-Sensitivity	Proportion of false positives in relation to all screenees
**Total number of attributes**	13	12	10	2	2	1	1	1	12	4	2	1	4
**Rating**													
Hawley et al., 2008 [46]	1^a^	1	1						1^b^				
**Ranking**													
Gyrd-Hansen et al., 2001 [58]		1											1^c^
**Discrete choice**													
**Generic**													
Salkeld et al., 2000 [55]			1										1^d^
Salkeld et al., 2003 [56]													1^e^
Marshall et al., 2007 [39]	1		1						1	1			
Howard et al., 2009 [43]	1		1						2	1			
Marshall et al., 2009 [47]	1^a^	1	1		1				1	1			
Van Dam et al., 2010 [36]		1	1	1		1							
Nayaradou et al., 2010 [59]	1						1		1				1^f^
Pignone et al., 2012 [44]	1^a^	1											
Brenner et al., 2014 [45]	1^a^	1			1								
Groothuis-Oudshoorn et al., 2014 [53]	1^a^	1	1						1	1			
Pignone et al., 2014 [48]	1												
Kistler et al., 2015 [49]	1^a^	1											
Martens et al., 2016 [50]	1												
Osborne et al., 2018 [57]	1								1^b^				
Mansfield et al., 2018 [51]			1					1	1^a^^,b^				
Ramezani_Doroh et al., 2019 [60]	1^a^	1	1	1					1				
De Bekker-Grob et al., 2019 [41]		1										1	
Phisalprapa et al., 2021 [61]		1	1										
**Labelled**													
Hol et al., 2010 [38]		1											
Benning et al., 2014 [52]									1		1		
Benning et al., 2014 [54]									1		1		

Categories	Benefits (*n* = 13)		Harms (*n* = 16)		Structural characteristics of health care (*n* = 24)					
Attributes	Reduction in colorectal cancer mortality (and incidence)	Colorectal cancer survival	Test-related pain and/or discomfort	Risk of complications	Out-of-pocket costs	Information processes	Travel time to screening facility	Waiting time for follow-up-test	Supervision	Level of evidence
**Total number of attributes**	12	1	8	8	15	5	2	1	1	2
**Rating**										
Hawley et al., 2008 [46]			1							
**Ranking**										
Gyrd-Hansen et al., 2001 [58]	1				1					
**Discrete choice**										
**Generic**										
Salkeld et al., 2000 [55]					1	1			1	
Salkeld et al., 2003 [56]	1					1				
Marshall et al., 2007 [39]			1		1					
Howard et al., 2009 [43]					1					
Marshall et al., 2009 [47]			1	1	1					
Van Dam et al., 2010 [36]	1		1	1						
Nayaradou et al., 2010 [59]	1				1	1				
Pignone et al., 2012 [44]	1^a^		1	1	1					
Brenner et al., 2014 [45]	1^a^			1						
Groothuis-Oudshoorn et al., 2014 [53]				1						
Pignone et al., 2014 [48]					2		1			
Kistler et al., 2015 [49]	1			1						
Martens et al., 2016 [50]					2		1			
Osborne et al., 2018 [57]					1					
Mansfield et al., 2018 [51]			1^a^		1					
Ramezani_Doroh et al., 2019 [60]	1		1	1	1					
De Bekker-Grob et al., 2019 [41]		1				1		1		
Phisalprapa et al., 2021 [61]	1		1	1	1					
**Labelled**										
Hol et al., 2010 [38]	1									
Benning et al., 2014 [52]	1									1
Benning et al., 2014 [54]	1					1				1

^a^Combination of different aspects within one attribute

^b^Stated as accuracy and/or performance by the authors

^c^Stated as risk of being called in for an unnecessary colonoscopy

^d^Stated as chance of a false positive test by the authors

^e^Stated as chance of a false positive test result and requiring an unnecessary colonoscopy by the authors

^f^Stated as the number of unnecessary colonoscopies generated by the possible occurrence of false-positive results by the authors

### Procedure-related characteristics

Twenty studies [36, 38, 39, 41, 43–51, 53, 55, 57–61] included at least one attribute assigned to procedure-related characteristics of CRC screening tests, with most of them (*n* = 14, [38, 39, 41, 43, 44, 48–51, 55, 57–59, 61]) presenting one or two attributes in this category. The most frequently used attributes addressed kind of procedure (*n* = 13, [39, 43–50, 53, 57, 59, 60]), frequency (*n* = 12, [36, 38, 41, 44–47, 49, 53, 58, 60, 61]), and preparation (*n* = 10, [36, 39, 43, 46, 47, 51, 53, 55, 60, 61]). In 7 studies, the procedure attribute was a combination of the procedure itself and at least one of the following aspects: location of screening (*n* = 5, [44, 45, 47, 49, 53]), preparation (*n* = 3, [44, 45, 49]), recovery time (*n* = 3, [44, 45, 49]), requirement of sedation (*n* = 3, [46, 53, 60]), and test-related pain and/or discomfort (*n* = 2, [45, 49]) (e.g., nature of the test – half day preparation time, invasive test in a medical facility, mild-moderate discomfort, 1 h recovery time [45]). Recovery time and requirement of sedation were not included as individual attributes by any study.

Six studies had attributes that refer to the location (*n* = 2, [36, 60]) and duration (*n* = 1, [36]) of screening, mode of test delivery (*n* = 1, [59]), purpose of screening, which means the ability to remove polyps or cancers (*n* = 1, [51]), and requirement of a follow-up test (*n* = 2, [45, 47]). These attributes were always included in addition to at least one of the three most frequently used procedure-related characteristics.

### Test characteristics

Fifteen studies included at least one attribute related to test characteristics of CRC screening methods with most (*n* = 14) having 1 (*n* = 8, [41, 46, 51, 55–58, 60]) or 2 (*n* = 6, [39, 47, 52–54, 59]) attributes from this category.

The most frequently used test characteristic attribute was sensitivity (*n* = 12, [39, 43, 46, 47, 51–54, 57, 59, 60]). Three studies [46, 51, 57] referred to their attributes as test accuracy and/or performance. Based on further information provided by the authors of included studies, they were categorised as sensitivity. Eleven studies included an attribute on specificity (*n* = 4, [39, 43, 47, 53]), 1-specificity (*n* = 2, [52, 54]), 1-sensitivity (*n* = 1, [41]) and the number of false positive test results in relation to all screened persons (*n* = 4, [55, 56, 58, 59]).

### Benefits

The most frequently used attribute related to benefits was reduction in colorectal cancer-specific mortality (*n* = 12, [36, 38, 44, 45, 49, 52, 54, 56, 58–61]). Two studies [44, 45] addressed the effect on CRC incidence, but only in combination with that on cancer-specific mortality. An attribute on CRC survival was considered in 1 study [41].

### Harms

Eleven studies [36, 39, 44–47, 49, 51, 53, 60, 61] included at least one attribute on harms directly associated with CRC screening methods. With 8 studies each, the two attributes of this category, test-related pain and/or discomfort [36, 39, 44, 46, 47, 51, 60, 61] and risk of complications [36, 44, 45, 47, 49, 53, 60, 61], were considered equally often. Five studies [36, 44, 47, 60, 61] used both attributes.

### Structural characteristics of health care

Sixteen studies [39, 41, 43, 44, 47, 48, 50, 51, 54–61] included at least one attribute from this category in the definition of their choice tasks. The most frequently used attribute (*n* = 15, [39, 43, 44, 47, 48, 50, 51, 55, 57–61]) was out-of-pocket costs. Seven studies implemented attributes addressing information processes (*n* = 5, [41, 54–56, 59]), travel time required to screening facility (*n* = 2, [48, 50]), waiting time required for a potential follow-up test (*n* = 1, [41]) and/or whether or not test administration was supervised by a general practitioner (n = 1, [55]). Information processes comprise attributes on informing about test results (*n* = 3, [55, 56, 59]) and a potential follow-up test (*n* = 1, [54]) as well as on waiting time required for test results (*n* = 1, [41]).

### Level of evidence

A level of evidence attribute was included by 2 studies [52, 54]. Both times it represented the strength of the available scientific evidence for the levels of sensitivity, chance of an unnecessary follow-up test and risk reduction.

### Relative importance of attributes

Twenty-one publications on 20 studies were included in the analysis of the relative importance of attributes. Two studies [54, 60] were excluded because the calculation of relative importance values was not possible due to missing reference values for the β coefficients of continuous attributes.

Six studies only reported attribute-level preference weights by subgroups (*n* = 2, [36, 47]), alternative-specific labels (*n* = 2, [38, 52]), classes from a latent class model (*n* = 1, [51]), and framing alternatives (*n* = 1, [43]). More than one relative importance ranking was calculated for each of them. In 4 of these studies [38, 43, 47, 51], the rankings for the most and second important attribute differed slightly from each other, which is why they were considered several times in the respective frequency analysis.

**Fig. 3:**
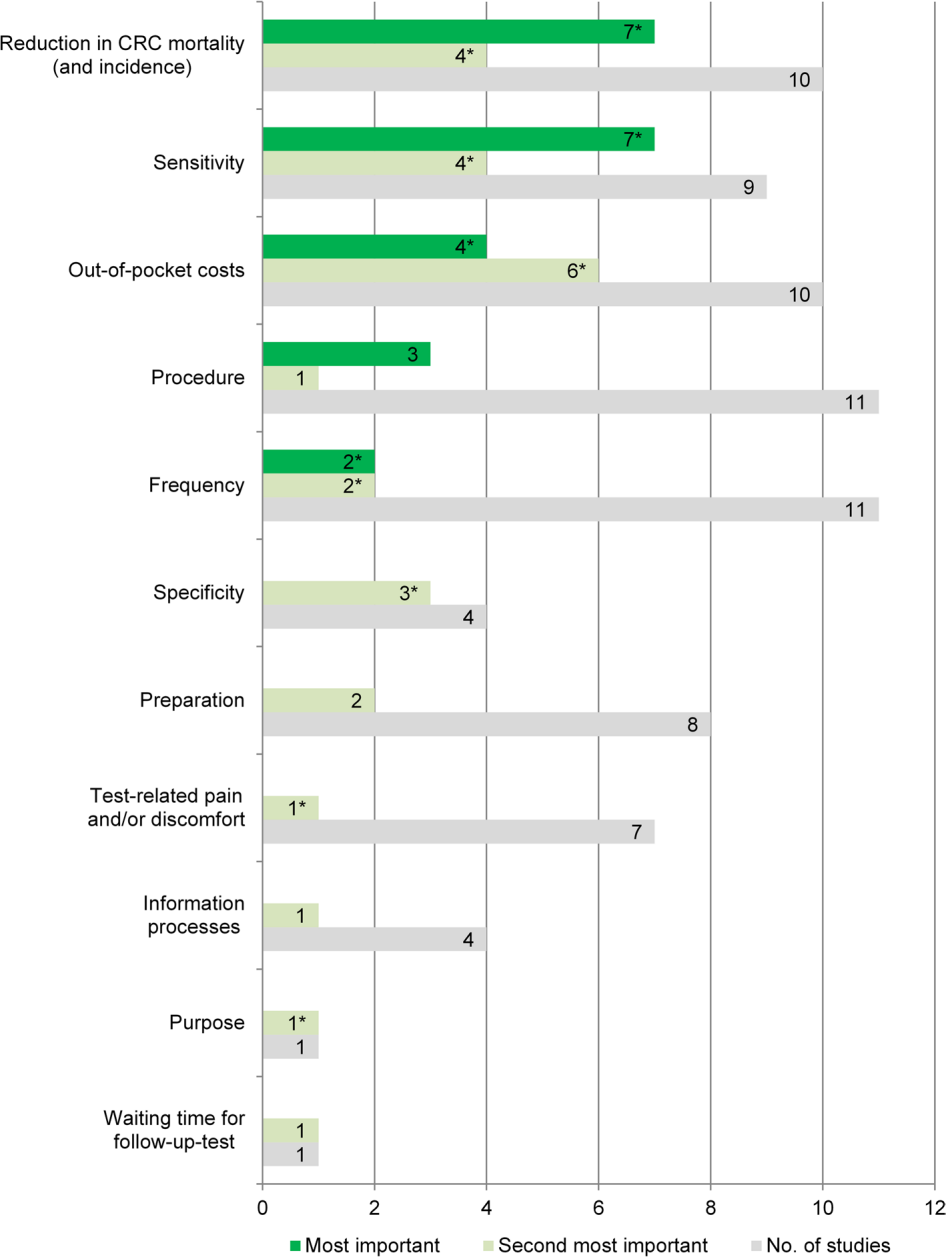
Most and second important attributes and number of studies with at least one of them. Legend: Results refer to 21 publications on 20 studies. * indicates that more than one most and/or second important attribute was extracted from each of 4 studies due to different relative importance rankings

Most important attributes were (in descending order) reduction in CRC mortality (and incidence) (*n* = 7, [36, 38, 44, 45, 56, 58, 61]), sensitivity (*n* = 7 [39, 43, 47, 51, 52, 57, 59],), out-of-pocket costs (*n* = 4 [48, 50, 51, 55],), kind of procedure (*n* = 3 [46, 49, 53],), and frequency (*n* = 2 [38, 41],) (Fig. 3 and Additional file 6 for relative importance of attributes per study). In addition, out-of-pocket costs (*n* = 6, [48, 50, 51, 57, 58, 61]), reduction in CRC mortality (and incidence) (*n* = 4, [38, 49, 52, 59]), and sensitivity (*n* = 4, [43, 46, 51, 53]) were most often ranked as second in importance for decision-making. Reduction in CRC mortality (and incidence) was not rated lower than second important in any study. With the exception of 2 studies, this also applies to sensitivity: This attribute was ranked as less important for one of three latent classes in Mansfield et al. [51] and for one of four frames in Howard et al. [43].

## Discussion

We identified 120 attributes from 23 publications on 22 studies and assigned them to six categories: procedure-related characteristics (*n* = 42; 35%), structural characteristics of health care (*n* = 24; 20%), test characteristics (*n* = 23; 19%), harms (*n* = 16; 13%), benefits (*n* = 13; 11%), and level of evidence (*n* = 2; 2%). The most frequently applied attributes in the choice tasks were out-of-pocket costs (*n* = 15), kind of procedure (*n* = 13), sensitivity (*n* = 12), test frequency (*n* = 12), reduction in CRC mortality (and incidence) (*n* = 12), preparation (*n* = 10), test-related pain and/or discomfort (*n* = 8), and risk of complications (*n* = 8). The calculated relative importance of attributes based on 21 publications of 20 studies discovered reduction in CRC mortality (and incidence) (*n* = 7), sensitivity (*n* = 7), out-of-pocket costs (*n* = 4), kind of procedure (*n* = 3), and frequency (*n* = 2) as being most important in decisions regarding screening for CRC.

One strength of our systematic review compared to others [10, 11, 21–24] is the risk of bias rating of the included studies using an adapted GRADE approach [34]. Overall risk of bias was rated as serious or critical in all but two of the studies reviewed, and was mainly due to deficiencies in the selection of participants (e.g., non-probability sampling methods like opt-in panels, convenience and purposeful approaches) and completeness of data (e.g., low response rates and lack of non-responder analyses). While these are no methodological weaknesses related to the construction, design and implementation of CAs or DCEs in particular, it may be of importance for the conduct of future stated preference elicitation studies. In 2011, the ISPOR (International Society for Pharmacoeconomics and Outcomes Research) Good Research Practices for Conjoint Analysis Task Force developed and published a checklist for conjoint analysis applications in health [7]. Consequently, for studies conducted after that point of time low risk of bias ratings were expected for the two subdomains measurement instrument and data analysis. However, none of the studies were rated with a low risk of bias for the measurement instrument subdomain at all; eleven had a serious or critical risk of bias due to shortcomings in a valid presentation of the outcome and in testing the understanding of the instrument, 7 of which were published after 2012. For the data analysis subdomain, a total of 10 studies were assessed with a low risk of bias, but 9 of these were published before 2011. In contrast, 9 out of 10 studies rated with a serious or critical risk of bias due to insufficiently accounting for preference heterogeneity in modelling were published in 2012 or later. This finding highlights the need for further implementation of the ISPOR checklist when conducting stated preference elicitation studies.

Among the 14 studies examining preferences for endoscopic screening methods, an attribute related to the reduction of colorectal cancer-specific incidence was identified only two times. In both cases, the effect on CRC incidence was only described in combination with colorectal cancer-specific mortality, but not as a single attribute. However, compound attributes are not recommended, because they increase the level of complexity and, at the same time, reduce information about which of the aspects primarily drives the choices, though frequently used [7, 23]. While stool-based tests can indirectly reduce the incidence of CRC via endoscopic follow-up, colonoscopy and sigmoidoscopy are able to directly prevent cancer by removing precancerous lesions [62]. Therefore, the extent of incidence reduction is an attribute in which CRC screening tests differ [63]. Moreover, the effect on disease-specific incidence qualifies as an even more relevant benefit outcome than disease-specific mortality [62, 64].

In line with previous reviews, sensitivity was identified as the most frequently used test characteristic attribute in the definition of preference elicitation tasks and led the relative most important rating among others [10, 11, 21, 23]. Taking into account recommendations on informed decision-making, this is surprising in several ways [65–67], as information on sensitivity (and also specificity) is not considered as appropriate and relevant for individual decisions in (cancer) screening [30]. Research has consistently shown that conditional probabilities like sensitivity and specificity have a high potential to be misunderstood by both consumers of health care and clinicians [68–71]. In addition, sensitivity allows no inference to the overall benefits or harms associated with the test, e.g., despite a high sensitivity there will be a high probability for false positive test results if the disease in question has a low prevalence as is the case in cancers [72]. To enable informed decision-making in the context of screening, presenting probabilities as natural frequencies and providing information on at least the baseline risk of the condition of interest and on both the probability of false negatives and false positives, which are considered patient-important outcomes due to inaccurate test results, are recommended [71, 73–75]. Studies eliciting preferences should incorporate current research findings in informed decision-making.

Our results further demonstrate that only 13 (57%) and 11 (48%) of the reviewed studies included an attribute addressing benefits and potential harms associated with CRC screening tests, respectively. Both a benefit- and a harms-related outcome were used in only 6 (26%) studies. This is notable, as informed decision-making requires a balanced presentation of benefits and harms [27, 28, 76, 77]. Our findings are, however, consistent with the results of Caverly et al. [78], who evaluated the presentation of benefits and harms in (colorectal) cancer screening recommendations and found that 25% (*n* = 14) and 29% (*n* = 16) of 55 positive recommendation statements from 32 guideline documents did not mention clinically important benefits or harms of cancer prevention at all.

We are aware that our systematic review has limitations. First, our results should be interpreted with caution because the relative importance of an attribute depends on both the range of levels and the other attributes included to describe the respective preference elicitation task [9, 33]. Therefore, attribute importance can only be analysed appropriately relative to the other attributes within the same choice experiment. However, the reviewed studies were highly heterogeneous in the kind and number of attributes as well as in the level ranges of similar attributes included. Consequently, a comparison of the relative importance of attributes across studies can only be approximate. Second, the results of the included studies may have limited validity due to their risk of bias, which may also affect this review’s conclusions. Finally, although we developed our search strategy taking into account the Cochrane Handbook for Systematic Reviews of Interventions [25] and the PRISMA Statement [26], it is possible that additional relevant studies could have been found by searching study registries, using other search terms, including non-English language studies, or publications issued before 2000.

## Conclusions

Stated preference elicitation methods have often been used to evaluate preferences of the screening eligible population for CRC screening. The risk of bias assessment revealed weaknesses in included studies, particularly in the selection of participants and completeness of data, and to a lesser extent in the measurement instrument and data analysis. To enhance study quality of future stated preference elicitation studies, the use of random sampling, analysis of differences between responders and non-responders in cases of low response rates, and adherence to the ISPOR checklist are recommended. We rated the risk of bias using a GRADE approach adapted to the methodological standards of DCEs and CAs. To obtain valuable feedback on the adaptation’s applicability, we welcome further use by other researchers.

While procedure-related attributes were most frequently used in the definition of choice tasks, reduction in colorectal cancer-specific mortality (and incidence) and sensitivity achieved high relative importance by screenees. A deeper analysis of the compositions of choice tasks revealed the following deficits. 1) Attributes on benefits and harms were used in an unbalanced way and were missing in almost half of the choice tasks, 2) attributes being inappropriate for individual decisions regarding screening, e.g., sensitivity, were included, and 3) a highly relevant benefit associated attribute for consumers, e.g., cancer-specific incidence reduction, was often lacking. In future stated preference elicitation studies, the identification and selection of attributes should be based both on evidence resulting from literature reviews and qualitative research reflecting consumers’ perspective, and on the principles of informed decision-making; especially in cases where preferences of screenees are evaluated to inform regulatory decisions in health care.

## Supplementary Information

**Additional file 1.** PRISMA Checklist

**Additional file 2.** Search terms used in PubMed and full electronic search strategy applied to the databases

**Additional file 3.** Risk of bias subdomains and signalling questions based on the approach addressing the certainty of evidence in the relative importance of outcomes or values and preferences developed by GRADE, adapted to conjoint analyses and discrete choice experiments

**Additional file 4.** List of records excluded due to full-text screening and primary reasons for exclusion

**Additional file 5.** Consensus answers and ratings of risk of bias including free-text support and direct quotations for included discrete choice experiments and conjoint analyses

**Additional file 6.** Calculated relative importance of attributes per study

## Data Availability

All data generated or analysed during this study are included in this published article and its supplementary information files.

## Abbreviations

CA: Conjoint analysis
CRC: Colorectal cancer
DCE: Discrete choice experiment
FDA: U.S. Food and Drug Administration
FIT: Faecal immunochemical test
FOBT: Faecal occult blood testing
gFOBT: Guaiac-based faecal occult blood testing
GRADE: Grading of Recommendations Assessment, Development and Evaluation
ISPOR: International Society for Pharmacoeconomics and Outcomes Research
PRISMA: Preferred Reporting Items for Systematic reviews and Meta-Analyses
SIGMO: Sigmoidoscopy as an evidence-based colorectal cancer screening test – a possible option?
