# Supplementary material for: Attributes in stated preference elicitation studies on colorectal cancer screening and their relative importance for decision-making among screenees: a systematic review
Source: Health Econ Rev. 2022 Sep 22;12:49. doi: 10.1186/s13561-022-00394-8 (PMC9494881; doi:10.1186/s13561-022-00394-8)
Supplement: Supplementary file 2 — Additional file 2. Search terms used in PubMed and full electronic search strategy applied to the databases [file 13561_2022_394_MOESM2_ESM.pdf]

Additional file 2 Search terms used in PubMed[a] and full electronic search strategy applied to the databases

[Screening test	OR	[Colorectal cancer	AND	Screening]	OR	Colorectal cancer screening]	AND	Preferences
**Free-text terms**								
**OR**		**OR**		**OR**		**OR**		**OR**
Colonoscop*		Colorectal cancer*		Screening*		Bowel cancer screening*		Conjoint analys*
Coloscop*		Colon cancer*		Early detection		Colorectal Screening*		Conjoint-analys*
Sigmoidoscop*		Bowel cancer*		Early diagnosis		Colorectal cancer screening*		Conjoint study
Stool test*		Rectal cancer*				Colorectal Prevention*		Conjoint studies
Fecal occult blood test*						Colorectal cancer prevention*		Conjoint measurement*
Faecal occult blood test*						Bowel cancer testing program*		Conjoint-measurement*
Faecal immunochemical test*						CRC screening*		Discrete choice*
Fecal immunochemical test*								Discrete-choice*
								Discrete rank*
								Ranking*
								Ranking exercise*
								Rating*
								Rating scale*
								Stated preference*
								Stated-preference*
								Patient preference*
								Patients preference*
								Patient's preference*
								Patients' preference*
								Patient attitude*
								Patients attitude*
								Patient's attitude*
								Patients' attitude*
								Patient choice*
								Patients choice*
								Patient's choice*
								Patients' choice*
								Choice based
								Choice-based
								Public preference*
								Health priorit*
**Controlled vocabulary search terms (MeSH terms)**								
**OR**		**OR**		**OR**		**OR**		**OR**
Colonoscopy		Colorectal Neoplasms		Mass Screening				Choice behavior
Sigmoidoscopy		Rectal Neoplasms		Early Diagnosis				Attitude to Health
				Early Detection of Cancer				Patient preference
								Consumer behavior
								Decision making

[a]Our search was database specific restricted to the search fields title, abstract and author keywords

PubMed		
**Initial search**		
– Most recent date of search: 15 July 2019		
– Date range of search: January 2000 to June 2019		
**No.**	**Query**	**Items found**
#73	Search (((((((((((((((((colonoscop*[Title/Abstract] AND ("2000/01/01"[PDat] : "2019/07/15"[PDat]))) OR (coloscop*[Title/Abstract] AND ("2000/01/01"[PDat] : "2019/07/15"[PDat]))) OR (sigmoidoscop*[Title/Abstract] AND ("2000/01/01"[PDat] : "2019/07/15"[PDat]))) OR (stool test*[Title/Abstract] AND ("2000/01/01"[PDat] : "2019/07/15"[PDat]))) OR (Fecal Occult Blood Test*[Title/Abstract] AND ("2000/01/01"[PDat] : "2019/07/15"[PDat]))) OR (Faecal Occult blood test*[Title/Abstract] AND ("2000/01/01"[PDat] : "2019/07/15"[PDat]))) OR (Faecal immunochemical test*[Title/Abstract] AND ("2000/01/01"[PDat] : "2019/07/15"[PDat]))) OR (Fecal immunochemical test*[Title/Abstract] AND ("2000/01/01"[PDat] : "2019/07/15"[PDat]))) OR (Colonoscopy[MeSH Terms] AND ("2000/01/01"[PDat] : "2019/07/15"[PDat]))) OR (Sigmoidoscopy[MeSH Terms] AND ("2000/01/01"[PDat] : "2019/07/15"[PDat]))) AND ("2000/01/01"[PDat] : "2019/07/15"[PDat]))) OR (((((((((((colorectal cancer*[Title/Abstract] AND ("2000/01/01"[PDat] : "2019/07/15"[PDat]))) OR (colon cancer*[Title/Abstract] AND ("2000/01/01"[PDat] : "2019/07/15"[PDat]))) OR (bowel cancer*[Title/Abstract] AND ("2000/01/01"[PDat] : "2019/07/15"[PDat]))) OR (rectal cancer*[Title/Abstract] AND ("2000/01/01"[PDat] : "2019/07/15"[PDat]))) OR (Colorectal Neoplasms[MeSH Terms] AND ("2000/01/01"[PDat] : "2019/07/15"[PDat]))) OR (Rectal Neoplasms[MeSH Terms] AND ("2000/01/01"[PDat] : "2019/07/15"[PDat]))) AND ("2000/01/01"[PDat] : "2019/07/15"[PDat]))) AND ((((((((screening*[Title/Abstract] AND ("2000/01/01"[PDat] : "2019/07/15"[PDat]))) OR (early detection[Title/Abstract] AND ("2000/01/01"[PDat] : "2019/07/15"[PDat]))) OR (early diagnosis[Title/Abstract] AND ("2000/01/01"[PDat] : "2019/07/15"[PDat]))) OR (Mass Screening[MeSH Terms] AND ("2000/01/01"[PDat] : "2019/07/15"[PDat]))) OR (Early Diagnosis[MeSH Terms] AND ("2000/01/01"[PDat] : "2019/07/15"[PDat]))) OR (Early Detection of Cancer[MeSH Terms] AND ("2000/01/01"[PDat] : "2019/07/15"[PDat]))) AND ("2000/01/01"[PDat] : "2019/07/15"[PDat]))) AND ("2000/01/01"[PDat] : "2019/07/15"[PDat]))) OR (((((((((bowel cancer screening*[Title/Abstract] AND ("2000/01/01"[PDat] : "2019/07/15"[PDat]))) OR (Colorectal Screening*[Title/Abstract] AND ("2000/01/01"[PDat] : "2019/07/15"[PDat]))) OR (colorectal cancer screening*[Title/Abstract] AND ("2000/01/01"[PDat] : "2019/07/15"[PDat]))) OR (Colorectal Prevention*[Title/Abstract] AND ("2000/01/01"[PDat] : "2019/07/15"[PDat]))) OR (Colorectal cancer prevention*[Title/Abstract] AND ("2000/01/01"[PDat] : "2019/07/15"[PDat]))) OR (bowel cancer testing program*[Title/Abstract] AND ("2000/01/01"[PDat] : "2019/07/15"[PDat]))) OR (CRC screening*[Title/Abstract] AND ("2000/01/01"[PDat] : "2019/07/15"[PDat]))) AND ("2000/01/01"[PDat] : "2019/07/15"[PDat]))) AND ("2000/01/01"[PDat] : "2019/07/15"[PDat]))) AND (((((((((((((((((((((((((((((((((((((CONJOINT ANALYS*[Title/Abstract] AND ("2000/01/01"[PDat] : "2019/07/15"[PDat]))) OR (CONJOINT-ANALYS*[Title/Abstract] AND ("2000/01/01"[PDat] : "2019/07/15"[PDat]))) OR (CONJOINT STUDY[Title/Abstract] AND ("2000/01/01"[PDat] : "2019/07/15"[PDat]))) OR (CONJOINT STUDIES[Title/Abstract] AND ("2000/01/01"[PDat] : "2019/07/15"[PDat]))) OR (conjoint measurement*[Title/Abstract] AND ("2000/01/01"[PDat] : "2019/07/15"[PDat]))) OR (conjoint-measurement*[Title/Abstract] AND ("2000/01/01"[PDat] : "2019/07/15"[PDat]))) OR (DISCRETE CHOICE*[Title/Abstract] AND ("2000/01/01"[PDat] : "2019/07/15"[PDat]))) OR (DISCRETE-CHOICE*[Title/Abstract] AND ("2000/01/01"[PDat] : "2019/07/15"[PDat]))) OR (DISCRETE RANK*[Title/Abstract] AND ("2000/01/01"[PDat] : "2019/07/15"[PDat]))) OR (ranking*[Title/Abstract] AND ("2000/01/01"[PDat] : "2019/07/15"[PDat]))) OR (ranking exercise*[Title/Abstract] AND ("2000/01/01"[PDat] : "2019/07/15"[PDat]))) OR (rating*[Title/Abstract] AND ("2000/01/01"[PDat] : "2019/07/15"[PDat]))) OR (rating scale*[Title/Abstract] AND ("2000/01/01"[PDat] : "2019/07/15"[PDat]))) OR (STATED PREFERENCE*[Title/Abstract] AND ("2000/01/01"[PDat] : "2019/07/15"[PDat]))) OR (STATED-PREFERENCE*[Title/Abstract] AND ("2000/01/01"[PDat] : "2019/07/15"[PDat]))) OR (PATIENT PREFERENCE*[Title/Abstract] AND ("2000/01/01"[PDat] : "2019/07/15"[PDat]))) OR (PATIENTS PREFERENCE*[Title/Abstract] AND ("2000/01/01"[PDat] : "2019/07/15"[PDat]))) OR (PATIENT'S PREFERENCE*[Title/Abstract] AND ("2000/01/01"[PDat] : "2019/07/15"[PDat]))) OR (PATIENTS' PREFERENCE*[Title/Abstract] AND ("2000/01/01"[PDat] : "2019/07/15"[PDat]))) OR (PATIENT ATTITUDE*[Title/Abstract] AND ("2000/01/01"[PDat] : "2019/07/15"[PDat]))) OR (PATIENTS ATTITUDE*[Title/Abstract] AND ("2000/01/01"[PDat] : "2019/07/15"[PDat]))) OR (PATIENT'S ATTITUDE*[Title/Abstract] AND ("2000/01/01"[PDat] : "2019/07/15"[PDat]))) OR (PATIENTS' ATTITUDE*[Title/Abstract] AND ("2000/01/01"[PDat] : "2019/07/15"[PDat]))) OR (Patient Choice*[Title/Abstract] AND ("2000/01/01"[PDat] : "2019/07/15"[PDat]))) OR (Patients Choice*[Title/Abstract] AND ("2000/01/01"[PDat] : "2019/07/15"[PDat]))) OR (Patient's Choice*[Title/Abstract] AND ("2000/01/01"[PDat] : "2019/07/15"[PDat]))) OR (Patients' Choice*[Title/Abstract] AND ("2000/01/01"[PDat] : "2019/07/15"[PDat]))) OR (choice based[Title/Abstract] AND ("2000/01/01"[PDat] : "2019/07/15"[PDat]))) OR (choice-based[Title/Abstract] AND ("2000/01/01"[PDat] : "2019/07/15"[PDat]))) OR (PUBLIC	**3,850**

	PREFERENCE*[Title/Abstract] AND ("2000/01/01"[PDat] : "2019/07/15"[PDat]))) OR (HEALTH PRIORIT*[Title/Abstract] AND ("2000/01/01"[PDat] : "2019/07/15"[PDat]))) OR (choice behavior[MeSH Terms] AND ("2000/01/01"[PDat] : "2019/07/15"[PDat]))) OR (Attitude to Health[MeSH Terms] AND ("2000/01/01"[PDat] : "2019/07/15"[PDat]))) OR (patient preference[MeSH Terms] AND ("2000/01/01"[PDat] : "2019/07/15"[PDat]))) OR (consumer behavior[MeSH Terms] AND ("2000/01/01"[PDat] : "2019/07/15"[PDat]))) OR (decision making[MeSH Terms] AND ("2000/01/01"[PDat] : "2019/07/15"[PDat]))) AND ("2000/01/01"[PDat] : "2019/07/15"[PDat])) Filters: Publication date from 2000/01/01 to 2019/07/15	
#72	Search ((((((((((((((((((((((((((((((((((CONJOINT ANALYS*[Title/Abstract] AND ("2000/01/01"[PDat] : "2019/07/15"[PDat]))) OR (CONJOINT-ANALYS*[Title/Abstract] AND ("2000/01/01"[PDat] : "2019/07/15"[PDat]))) OR (CONJOINT STUDY[Title/Abstract] AND ("2000/01/01"[PDat] : "2019/07/15"[PDat]))) OR (CONJOINT STUDIES[Title/Abstract] AND ("2000/01/01"[PDat] : "2019/07/15"[PDat]))) OR (conjoint measurement*[Title/Abstract] AND ("2000/01/01"[PDat] : "2019/07/15"[PDat]))) OR (conjoint-measurement*[Title/Abstract] AND ("2000/01/01"[PDat] : "2019/07/15"[PDat]))) OR (DISCRETE CHOICE*[Title/Abstract] AND ("2000/01/01"[PDat] : "2019/07/15"[PDat]))) OR (DISCRETE-CHOICE*[Title/Abstract] AND ("2000/01/01"[PDat] : "2019/07/15"[PDat]))) OR (DISCRETE RANK*[Title/Abstract] AND ("2000/01/01"[PDat] : "2019/07/15"[PDat]))) OR (ranking*[Title/Abstract] AND ("2000/01/01"[PDat] : "2019/07/15"[PDat]))) OR (ranking exercise*[Title/Abstract] AND ("2000/01/01"[PDat] : "2019/07/15"[PDat]))) OR (rating*[Title/Abstract] AND ("2000/01/01"[PDat] : "2019/07/15"[PDat]))) OR (rating scale*[Title/Abstract] AND ("2000/01/01"[PDat] : "2019/07/15"[PDat]))) OR (STATED PREFERENCE*[Title/Abstract] AND ("2000/01/01"[PDat] : "2019/07/15"[PDat]))) OR (STATED-PREFERENCE*[Title/Abstract] AND ("2000/01/01"[PDat] : "2019/07/15"[PDat]))) OR (PATIENT PREFERENCE*[Title/Abstract] AND ("2000/01/01"[PDat] : "2019/07/15"[PDat]))) OR (PATIENTS PREFERENCE*[Title/Abstract] AND ("2000/01/01"[PDat] : "2019/07/15"[PDat]))) OR (PATIENT'S PREFERENCE*[Title/Abstract] AND ("2000/01/01"[PDat] : "2019/07/15"[PDat]))) OR (PATIENTS' PREFERENCE*[Title/Abstract] AND ("2000/01/01"[PDat] : "2019/07/15"[PDat]))) OR (PATIENT ATTITUDE*[Title/Abstract] AND ("2000/01/01"[PDat] : "2019/07/15"[PDat]))) OR (PATIENTS ATTITUDE*[Title/Abstract] AND ("2000/01/01"[PDat] : "2019/07/15"[PDat]))) OR (PATIENT'S ATTITUDE*[Title/Abstract] AND ("2000/01/01"[PDat] : "2019/07/15"[PDat]))) OR (PATIENTS' ATTITUDE*[Title/Abstract] AND ("2000/01/01"[PDat] : "2019/07/15"[PDat]))) OR (Patient Choice*[Title/Abstract] AND ("2000/01/01"[PDat] : "2019/07/15"[PDat]))) OR (Patients Choice*[Title/Abstract] AND ("2000/01/01"[PDat] : "2019/07/15"[PDat]))) OR (Patient's Choice*[Title/Abstract] AND ("2000/01/01"[PDat] : "2019/07/15"[PDat]))) OR (Patients' Choice*[Title/Abstract] AND ("2000/01/01"[PDat] : "2019/07/15"[PDat]))) OR (choice based[Title/Abstract] AND ("2000/01/01"[PDat] : "2019/07/15"[PDat]))) OR (choice-based[Title/Abstract] AND ("2000/01/01"[PDat] : "2019/07/15"[PDat]))) OR (PUBLIC PREFERENCE*[Title/Abstract] AND ("2000/01/01"[PDat] : "2019/07/15"[PDat]))) OR (HEALTH PRIORIT*[Title/Abstract] AND ("2000/01/01"[PDat] : "2019/07/15"[PDat]))) OR (choice behavior[MeSH Terms] AND ("2000/01/01"[PDat] : "2019/07/15"[PDat]))) OR (Attitude to Health[MeSH Terms] AND ("2000/01/01"[PDat] : "2019/07/15"[PDat]))) OR (patient preference[MeSH Terms] AND ("2000/01/01"[PDat] : "2019/07/15"[PDat]))) OR (consumer behavior[MeSH Terms] AND ("2000/01/01"[PDat] : "2019/07/15"[PDat]))) OR (decision making[MeSH Terms] AND ("2000/01/01"[PDat] : "2019/07/15"[PDat])) Filters: Publication date from 2000/01/01 to 2019/07/15	563546
#71	Search decision making[MeSH Terms] Filters: Publication date from 2000/01/01 to 2019/07/15	137739
#70	Search consumer behavior[MeSH Terms] Filters: Publication date from 2000/01/01 to 2019/07/15	10267
#69	Search patient preference[MeSH Terms] Filters: Publication date from 2000/01/01 to 2019/07/15	7327
#68	Search Attitude to Health[MeSH Terms] Filters: Publication date from 2000/01/01 to 2019/07/15	302023
#67	Search choice behavior[MeSH Terms] Filters: Publication date from 2000/01/01 to 2019/07/15	41136
#66	Search HEALTH PRIORIT*[Title/Abstract] Filters: Publication date from 2000/01/01 to 2019/07/15	3631
#65	Search PUBLIC PREFERENCE*[Title/Abstract] Filters: Publication date from 2000/01/01 to 2019/07/15	223
#64	Search choice-based[Title/Abstract] Filters: Publication date from 2000/01/01 to 2019/07/15	464
#63	Search choice based[Title/Abstract] Filters: Publication date from 2000/01/01 to 2019/07/15	464
#62	Search Patients' Choice*[Title/Abstract] Filters: Publication date from 2000/01/01 to 2019/07/15	238
#61	Search Patient's Choice*[Title/Abstract] Filters: Publication date from 2000/01/01 to 2019/07/15	325
#60	Search Patients Choice*[Title/Abstract] Filters: Publication date from 2000/01/01 to 2019/07/15	280
#59	Search Patient Choice*[Title/Abstract] Filters: Publication date from 2000/01/01 to 2019/07/15	1538

#58	Search PATIENTS' ATTITUDE*[Title/Abstract] Filters: Publication date from 2000/01/01 to 2019/07/15	952
#57	Search PATIENT'S ATTITUDE*[Title/Abstract] Filters: Publication date from 2000/01/01 to 2019/07/15	171
#56	Search PATIENTS ATTITUDE*[Title/Abstract] Filters: Publication date from 2000/01/01 to 2019/07/15	997
#55	Search PATIENT ATTITUDE*[Title/Abstract] Filters: Publication date from 2000/01/01 to 2019/07/15	750
#54	Search PATIENTS' PREFERENCE*[Title/Abstract] Filters: Publication date from 2000/01/01 to 2019/07/15	2079
#53	Search PATIENT'S PREFERENCE*[Title/Abstract] Filters: Publication date from 2000/01/01 to 2019/07/15	833
#52	Search PATIENTS PREFERENCE*[Title/Abstract] Filters: Publication date from 2000/01/01 to 2019/07/15	2175
#51	Search PATIENT PREFERENCE*[Title/Abstract] Filters: Publication date from 2000/01/01 to 2019/07/15	7254
#50	Search STATED-PREFERENCE*[Title/Abstract] Filters: Publication date from 2000/01/01 to 2019/07/15	619
#49	Search STATED PREFERENCE*[Title/Abstract] Filters: Publication date from 2000/01/01 to 2019/07/15	619
#48	Search rating scale*[Title/Abstract] Filters: Publication date from 2000/01/01 to 2019/07/15	44161
#47	Search rating*[Title/Abstract] Filters: Publication date from 2000/01/01 to 2019/07/15	114947
#46	Search ranking exercise*[Title/Abstract] Filters: Publication date from 2000/01/01 to 2019/07/15	99
#45	Search ranking*[Title/Abstract] Filters: Publication date from 2000/01/01 to 2019/07/15	20810
#44	Search DISCRETE RANK*[Title/Abstract] Filters: Publication date from 2000/01/01 to 2019/07/15	3
#43	Search DISCRETE-CHOICE*[Title/Abstract] Filters: Publication date from 2000/01/01 to 2019/07/15	1787
#42	Search DISCRETE CHOICE*[Title/Abstract] Filters: Publication date from 2000/01/01 to 2019/07/15	1787
#41	Search conjoint-measurement*[Title/Abstract] Filters: Publication date from 2000/01/01 to 2019/07/15	37
#40	Search conjoint measurement*[Title/Abstract] Filters: Publication date from 2000/01/01 to 2019/07/15	37
#39	Search CONJOINT STUDIES[Title/Abstract] Filters: Publication date from 2000/01/01 to 2019/07/15	2
#38	Search CONJOINT STUDY[Title/Abstract] Filters: Publication date from 2000/01/01 to 2019/07/15	13
#37	Search CONJOINT-ANALYS*[Title/Abstract] Filters: Publication date from 2000/01/01 to 2019/07/15	685
#36	Search CONJOINT ANALYS*[Title/Abstract] Filters: Publication date from 2000/01/01 to 2019/07/15	685
#35	Search (((((((((((((colonoscop*[Title/Abstract] AND ("2000/01/01"[PDat] : "2019/07/15"[PDat]))) OR (coloscop*[Title/Abstract] AND ("2000/01/01"[PDat] : "2019/07/15"[PDat]))) OR (sigmoidoscop*[Title/Abstract] AND ("2000/01/01"[PDat] : "2019/07/15"[PDat]))) OR (stool test*[Title/Abstract] AND ("2000/01/01"[PDat] : "2019/07/15"[PDat]))) OR (Fecal Occult Blood Test*[Title/Abstract] AND ("2000/01/01"[PDat] : "2019/07/15"[PDat]))) OR (Faecal Occult blood test*[Title/Abstract] AND ("2000/01/01"[PDat] : "2019/07/15"[PDat]))) OR (Faecal immunochemical test*[Title/Abstract] AND ("2000/01/01"[PDat] : "2019/07/15"[PDat]))) OR (Fecal immunochemical test*[Title/Abstract] AND ("2000/01/01"[PDat] : "2019/07/15"[PDat]))) OR (Colonoscopy[MeSH Terms] AND ("2000/01/01"[PDat] : "2019/07/15"[PDat]))) OR (Sigmoidoscopy[MeSH Terms] AND ("2000/01/01"[PDat] : "2019/07/15"[PDat]))) AND ("2000/01/01"[PDat] : "2019/07/15"[PDat]))) OR ((((((((((colorectal cancer*[Title/Abstract] AND ("2000/01/01"[PDat] : "2019/07/15"[PDat]))) OR (colon cancer*[Title/Abstract] AND ("2000/01/01"[PDat] : "2019/07/15"[PDat]))) OR (bowel cancer*[Title/Abstract] AND ("2000/01/01"[PDat] : "2019/07/15"[PDat]))) OR (rectal cancer*[Title/Abstract] AND ("2000/01/01"[PDat] : "2019/07/15"[PDat]))) OR (Colorectal Neoplasms[MeSH Terms] AND ("2000/01/01"[PDat] : "2019/07/15"[PDat]))) OR (Rectal Neoplasms[MeSH Terms] AND (	49228

	"2000/01/01"[PDat] : "2019/07/15"[PDat]))) AND ("2000/01/01"[PDat] : "2019/07/15"[PDat]))) AND ((((((((screening*[Title/Abstract] AND ("2000/01/01"[PDat] : "2019/07/15"[PDat]))) OR (early detection[Title/Abstract] AND ("2000/01/01"[PDat] : "2019/07/15"[PDat]))) OR (early diagnosis[Title/Abstract] AND ("2000/01/01"[PDat] : "2019/07/15"[PDat]))) OR (Mass Screening[MeSH Terms] AND ("2000/01/01"[PDat] : "2019/07/15"[PDat]))) OR (Early Diagnosis[MeSH Terms] AND ("2000/01/01"[PDat] : "2019/07/15"[PDat]))) OR (Early Detection of Cancer[MeSH Terms] AND ("2000/01/01"[PDat] : "2019/07/15"[PDat]))) AND ("2000/01/01"[PDat] : "2019/07/15"[PDat]))) AND ("2000/01/01"[PDat] : "2019/07/15"[PDat]))) OR (((((((((bowel cancer screening*[Title/Abstract] AND ("2000/01/01"[PDat] : "2019/07/15"[PDat]))) OR (Colorectal Screening*[Title/Abstract] AND ("2000/01/01"[PDat] : "2019/07/15"[PDat]))) OR (colorectal cancer screening*[Title/Abstract] AND ("2000/01/01"[PDat] : "2019/07/15"[PDat]))) OR (Colorectal Prevention*[Title/Abstract] AND ("2000/01/01"[PDat] : "2019/07/15"[PDat]))) OR (Colorectal cancer prevention*[Title/Abstract] AND ("2000/01/01"[PDat] : "2019/07/15"[PDat]))) OR (bowel cancer testing program*[Title/Abstract] AND ("2000/01/01"[PDat] : "2019/07/15"[PDat]))) OR (CRC screening*[Title/Abstract] AND ("2000/01/01"[PDat] : "2019/07/15"[PDat]))) AND ("2000/01/01"[PDat] : "2019/07/15"[PDat])) Filters: Publication date from 2000/01/01 to 2019/07/15	
#34	Search (((((((((colorectal cancer*[Title/Abstract] AND ("2000/01/01"[PDat] : "2019/07/15"[PDat]))) OR (colon cancer*[Title/Abstract] AND ("2000/01/01"[PDat] : "2019/07/15"[PDat]))) OR (bowel cancer*[Title/Abstract] AND ("2000/01/01"[PDat] : "2019/07/15"[PDat]))) OR (rectal cancer*[Title/Abstract] AND ("2000/01/01"[PDat] : "2019/07/15"[PDat]))) OR (Colorectal Neoplasms[MeSH Terms] AND ("2000/01/01"[PDat] : "2019/07/15"[PDat]))) OR (Rectal Neoplasms[MeSH Terms] AND ("2000/01/01"[PDat] : "2019/07/15"[PDat]))) AND ("2000/01/01"[PDat] : "2019/07/15"[PDat]))) AND ((((((((screening*[Title/Abstract] AND ("2000/01/01"[PDat] : "2019/07/15"[PDat]))) OR (early detection[Title/Abstract] AND ("2000/01/01"[PDat] : "2019/07/15"[PDat]))) OR (early diagnosis[Title/Abstract] AND ("2000/01/01"[PDat] : "2019/07/15"[PDat]))) OR (Mass Screening[MeSH Terms] AND ("2000/01/01"[PDat] : "2019/07/15"[PDat]))) OR (Early Diagnosis[MeSH Terms] AND ("2000/01/01"[PDat] : "2019/07/15"[PDat]))) OR (Early Detection of Cancer[MeSH Terms] AND ("2000/01/01"[PDat] : "2019/07/15"[PDat]))) AND ("2000/01/01"[PDat] : "2019/07/15"[PDat])) Filters: Publication date from 2000/01/01 to 2019/07/15	19560
#33	Search (((((((bowel cancer screening*[Title/Abstract] AND ("2000/01/01"[PDat] : "2019/07/15"[PDat]))) OR (Colorectal Screening*[Title/Abstract] AND ("2000/01/01"[PDat] : "2019/07/15"[PDat]))) OR (colorectal cancer screening*[Title/Abstract] AND ("2000/01/01"[PDat] : "2019/07/15"[PDat]))) OR (Colorectal Prevention*[Title/Abstract] AND ("2000/01/01"[PDat] : "2019/07/15"[PDat]))) OR (Colorectal cancer prevention*[Title/Abstract] AND ("2000/01/01"[PDat] : "2019/07/15"[PDat]))) OR (bowel cancer testing program*[Title/Abstract] AND ("2000/01/01"[PDat] : "2019/07/15"[PDat]))) OR (CRC screening*[Title/Abstract] AND ("2000/01/01"[PDat] : "2019/07/15"[PDat])) Filters: Publication date from 2000/01/01 to 2019/07/15	11775
#32	Search CRC screening*[Title/Abstract] Filters: Publication date from 2000/01/01 to 2019/07/15	2470
#31	Search bowel cancer testing program*[Title/Abstract] Filters: Publication date from 2000/01/01 to 2019/07/15	19
#30	Search Colorectal cancer prevention*[Title/Abstract] Filters: Publication date from 2000/01/01 to 2019/07/15	405
#29	Search Colorectal Prevention*[Title/Abstract] Filters: Publication date from 2000/01/01 to 2019/07/15	5861
#28	Search colorectal cancer screening*[Title/Abstract] Filters: Publication date from 2000/01/01 to 2019/07/15	5001
#27	Search Colorectal Screening*[Title/Abstract] Filters: Publication date from 2000/01/01 to 2019/07/15	422
#26	Search bowel cancer screening*[Title/Abstract] Filters: Publication date from 2000/01/01 to 2019/07/15	402
#25	Search ((((((screening*[Title/Abstract] AND ("2000/01/01"[PDat] : "2019/07/15"[PDat]))) OR (early detection[Title/Abstract] AND ("2000/01/01"[PDat] : "2019/07/15"[PDat]))) OR (early diagnosis[Title/Abstract] AND ("2000/01/01"[PDat] : "2019/07/15"[PDat]))) OR (Mass Screening[MeSH Terms] AND ("2000/01/01"[PDat] : "2019/07/15"[PDat]))) OR (Early Diagnosis[MeSH Terms] AND ("2000/01/01"[PDat] : "2019/07/15"[PDat]))) OR (Early Detection of Cancer[MeSH Terms] AND ("2000/01/01"[PDat] : "2019/07/15"[PDat])) Filters: Publication date from 2000/01/01 to 2019/07/15	508082
#24	Search Early Detection of Cancer[MeSH Terms] Filters: Publication date from 2000/01/01 to 2019/07/15	21353
#23	Search Early Diagnosis[MeSH Terms] Filters: Publication date from 2000/01/01 to 2019/07/15	43178

#22	Search Mass Screening[MeSH Terms] Filters: Publication date from 2000/01/01 to 2019/07/15	80783
#21	Search early diagnosis[Title/Abstract] Filters: Publication date from 2000/01/01 to 2019/07/15	52213
#20	Search early detection[Title/Abstract] Filters: Publication date from 2000/01/01 to 2019/07/15	44846
#19	Search screening*[Title/Abstract] Filters: Publication date from 2000/01/01 to 2019/07/15	389017
#18	Search ((((((colorectal cancer*[Title/Abstract] AND ("2000/01/01"[PDat] : "2019/07/15"[PDat]))) OR (colon cancer*[Title/Abstract] AND ("2000/01/01"[PDat] : "2019/07/15"[PDat]))) OR (bowel cancer*[Title/Abstract] AND ("2000/01/01"[PDat] : "2019/07/15"[PDat]))) OR (rectal cancer*[Title/Abstract] AND ("2000/01/01"[PDat] : "2019/07/15"[PDat]))) OR (Colorectal Neoplasms[MeSH Terms] AND ("2000/01/01"[PDat] : "2019/07/15"[PDat]))) OR (Rectal Neoplasms[MeSH Terms] AND ("2000/01/01"[PDat] : "2019/07/15"[PDat])) Filters: Publication date from 2000/01/01 to 2019/07/15	164462
#17	Search Rectal Neoplasms[MeSH Terms] Filters: Publication date from 2000/01/01 to 2019/07/15	21510
#16	Search Colorectal Neoplasms[MeSH Terms] Filters: Publication date from 2000/01/01 to 2019/07/15	126260
#15	Search rectal cancer*[Title/Abstract] Filters: Publication date from 2000/01/01 to 2019/07/15	17693
#14	Search bowel cancer*[Title/Abstract] Filters: Publication date from 2000/01/01 to 2019/07/15	1164
#13	Search colon cancer*[Title/Abstract] Filters: Publication date from 2000/01/01 to 2019/07/15	37797
#12	Search colorectal cancer*[Title/Abstract] Filters: Publication date from 2000/01/01 to 2019/07/15	82500
#11	Search (((((((((colonoscop*[Title/Abstract] AND ("2000/01/01"[PDat] : "2019/07/15"[PDat]))) OR (coloscop*[Title/Abstract] AND ("2000/01/01"[PDat] : "2019/07/15"[PDat]))) OR (sigmoidoscop*[Title/Abstract] AND ("2000/01/01"[PDat] : "2019/07/15"[PDat]))) OR (stool test*[Title/Abstract] AND ("2000/01/01"[PDat] : "2019/07/15"[PDat]))) OR (Fecal Occult Blood Test*[Title/Abstract] AND ("2000/01/01"[PDat] : "2019/07/15"[PDat]))) OR (Faecal Occult blood test*[Title/Abstract] AND ("2000/01/01"[PDat] : "2019/07/15"[PDat]))) OR (Faecal immunochemical test*[Title/Abstract] AND ("2000/01/01"[PDat] : "2019/07/15"[PDat]))) OR (Fecal immunochemical test*[Title/Abstract] AND ("2000/01/01"[PDat] : "2019/07/15"[PDat]))) OR (Colonoscopy[MeSH Terms] AND ("2000/01/01"[PDat] : "2019/07/15"[PDat]))) OR (Sigmoidoscopy[MeSH Terms] AND ("2000/01/01"[PDat] : "2019/07/15"[PDat])) Filters: Publication date from 2000/01/01 to 2019/07/15	34600
#10	Search Sigmoidoscopy[MeSH Terms] Filters: Publication date from 2000/01/01 to 2019/07/15	1953
#9	Search Colonoscopy[MeSH Terms] Filters: Publication date from 2000/01/01 to 2019/07/15	21221
#8	Search Fecal immunochemical test*[Title/Abstract] Filters: Publication date from 2000/01/01 to 2019/07/15	638
#7	Search Faecal immunochemical test*[Title/Abstract] Filters: Publication date from 2000/01/01 to 2019/07/15	242
#6	Search Faecal Occult blood test*[Title/Abstract] Filters: Publication date from 2000/01/01 to 2019/07/15	758
#5	Search Fecal Occult Blood Test*[Title/Abstract] Filters: Publication date from 2000/01/01 to 2019/07/15	1982
#4	Search stool test*[Title/Abstract] Filters: Publication date from 2000/01/01 to 2019/07/15	551
#3	Search sigmoidoscop*[Title/Abstract] Filters: Publication date from 2000/01/01 to 2019/07/15	2498
#2	Search coloscop*[Title/Abstract] Filters: Publication date from 2000/01/01 to 2019/07/15	142
#1	Search colonoscop*[Title/Abstract] Filters: Publication date from 2000/01/01 to 2019/07/15	23196

**Embase**	
**Initial search**	
– Most recent date of search: 15 July 2019	
– Date range of search: 2000 to July 2019	
**Query**	**Items found**
(colonoscop*:ti,ab,kw OR coloscop*:ti,ab,kw OR sigmoidoscop*:ti,ab,kw OR 'stool test*':ti,ab,kw OR 'fecal occult blood test*':ti,ab,kw OR 'faecal occult blood test*':ti,ab,kw OR 'faecal immunochemical test*':ti,ab,kw OR 'fecal immunochemical test*':ti,ab,kw OR 'colonoscopy'/exp OR 'sigmoidoscopy'/exp OR 'bowel cancer screening*':ti,ab,kw OR 'colorectal screening*':ti,ab,kw OR 'colorectal cancer screening*':ti,ab,kw OR 'colorectal prevention*':ti,ab,kw OR 'colorectal cancer prevention*':ti,ab,kw OR 'bowel cancer testing program*':ti,ab,kw OR 'crc screening*':ti,ab,kw OR (('colorectal cancer*':ti,ab,kw OR 'colon cancer*':ti,ab,kw OR 'bowel cancer*':ti,ab,kw OR 'rectal cancer*':ti,ab,kw OR 'colon tumor'/exp OR 'rectum tumor'/exp) AND (screening*:ti,ab,kw OR 'early detection':ti,ab,kw OR 'early diagnosis':ti,ab,kw OR 'mass screening'/exp OR 'early diagnosis'/exp OR 'early cancer diagnosis'/exp))) AND ('conjoint analys*':ti,ab,kw OR 'conjoint study':ti,ab,kw OR 'conjoint studies':ti,ab,kw OR 'conjoint measurement*':ti,ab,kw OR 'discrete choice*':ti,ab,kw OR 'discrete rank*':ti,ab,kw OR ranking*:ti,ab,kw OR 'ranking exercise*':ti,ab,kw OR rating*:ti,ab,kw OR 'rating scale*':ti,ab,kw OR 'stated preference*':ti,ab,kw OR 'patient preference*':ti,ab,kw OR 'patients preference*':ti,ab,kw OR 'patient attitude*':ti,ab,kw OR 'patients attitude*':ti,ab,kw OR 'patient choice*':ti,ab,kw OR 'patients choice*':ti,ab,kw OR 'choice based':ti,ab,kw OR 'public preference*':ti,ab,kw OR 'health priorit*':ti,ab,kw OR 'decision making'/exp OR 'attitude to health'/exp OR 'patient preference'/exp OR 'consumer attitude'/exp) AND (2000:py OR 2001:py OR 2002:py OR 2003:py OR 2004:py OR 2005:py OR 2006:py OR 2007:py OR 2008:py OR 2009:py OR 2010:py OR 2011:py OR 2012:py OR 2013:py OR 2014:py OR 2015:py OR 2016:py OR 2017:py OR 2018:py OR 2019:py)	**3,487**

Biomedical Reference Collection: Corporate (via EBSCO)	
– Most recent date of search: 15 July 2019	
– Date range of search: January 2000 to July 2019	
**Query**	**Items found**
(((TI colonoscop* OR AB colonoscop* OR KW colonoscop*) OR (TI coloscop* OR AB coloscop* OR KW coloscop*) OR (TI sigmoidoscop* OR AB sigmoidoscop* OR KW sigmoidoscop*) OR (TI stool test* OR AB stool test* OR KW stool test*) OR (TI Fecal Occult Blood Test* OR AB Fecal Occult Blood Test* OR KW Fecal Occult Blood Test*) OR (TI Faecal Occult blood test* OR AB Faecal Occult blood test* OR KW Faecal Occult blood test*) OR (TI Faecal immunochemical test* OR AB Faecal immunochemical test* OR KW Faecal immunochemical test*) OR (TI Fecal immunochemical test* OR AB Fecal immunochemical test* OR KW Fecal immunochemical test*) OR DE "COLONOSCOPY" OR DE "SIGMOIDOSCOPY") OR (((TI colorectal cancer* OR AB colorectal cancer* OR KW colorectal cancer*) OR (TI colon cancer* OR AB colon cancer* OR KW colon cancer*) OR (TI bowel cancer* OR AB bowel cancer* OR KW bowel cancer*) OR (TI rectal cancer* OR AB rectal cancer* OR KW rectal cancer*) OR (DE "COLON cancer" OR DE "HEREDITARY nonpolyposis colorectal cancer") OR DE "RECTAL cancer") AND ((TI screening* OR AB screening* OR KW screening*) OR (TI early detection OR AB early detection OR KW early detection) OR (TI early diagnosis OR AB early diagnosis OR KW early diagnosis) OR DE "MEDICAL screening" OR DE "EARLY diagnosis" OR DE "EARLY detection of cancer")) OR ((TI bowel cancer screening* OR AB bowel cancer screening* OR KW bowel cancer screening*) OR (TI Colorectal Screening* OR AB Colorectal Screening* OR KW Colorectal Screening*) OR (TI colorectal cancer screening* OR AB colorectal cancer screening* OR KW colorectal cancer screening*) OR (TI Colorectal Prevention* OR AB Colorectal Prevention* OR KW Colorectal Prevention*) OR (TI Colorectal cancer prevention* OR AB Colorectal cancer prevention* OR KW Colorectal cancer prevention*) OR (TI bowel cancer testing program* OR AB bowel cancer testing program* OR KW bowel cancer testing program*) OR (TI CRC screening* OR AB CRC screening* OR KW CRC screening*))) AND ((TI CONJOINT ANALYS* OR AB CONJOINT ANALYS* OR KW CONJOINT ANALYS*) OR (TI CONJOINT-ANALYS* OR AB CONJOINT-ANALYS* OR KW CONJOINT-ANALYS*) OR (TI CONJOINT STUDY OR AB CONJOINT STUDY OR KW CONJOINT STUDY) OR (TI CONJOINT STUDIES OR AB CONJOINT STUDIES OR KW CONJOINT STUDIES) OR (TI conjoint measurement* OR AB conjoint measurement* OR KW conjoint measurement*) OR (TI conjoint-measurement* OR AB conjoint-measurement* OR KW conjoint-measurement*) OR (TI DISCRETE CHOICE* OR AB DISCRETE CHOICE* OR KW DISCRETE CHOICE*) OR (TI DISCRETE-CHOICE* OR AB DISCRETE-CHOICE* OR KW DISCRETE-CHOICE*) OR (TI DISCRETE RANK* OR AB DISCRETE RANK* OR KW DISCRETE RANK*) OR (TI ranking* OR AB ranking* OR KW ranking*) OR (TI ranking exercise* OR AB ranking exercise* OR KW ranking exercise*) OR (TI rating* OR AB rating* OR KW rating*) OR (TI rating scale* OR AB rating scale* OR KW rating scale*) OR (TI STATED PREFERENCE* OR AB STATED PREFERENCE* OR KW STATED PREFERENCE*) OR (TI STATED-PREFERENCE* OR AB STATED-PREFERENCE* OR KW STATED-PREFERENCE*) OR (TI PATIENT PREFERENCE* OR AB PATIENT PREFERENCE* OR KW PATIENT PREFERENCE*) OR (TI PATIENTS PREFERENCE* OR AB PATIENTS PREFERENCE* OR KW PATIENTS PREFERENCE*) OR (TI PATIENT'S PREFERENCE* OR AB PATIENT'S PREFERENCE* OR KW PATIENT'S PREFERENCE*) OR (TI PATIENTS' PREFERENCE* OR AB PATIENTS' PREFERENCE* OR KW PATIENTS' PREFERENCE*) OR (TI PATIENT ATTITUDE* OR AB PATIENT ATTITUDE* OR KW PATIENT ATTITUDE*) OR (TI PATIENTS ATTITUDE* OR AB PATIENTS ATTITUDE* OR KW PATIENTS ATTITUDE*) OR (TI PATIENT'S ATTITUDE* OR AB PATIENT'S ATTITUDE* OR KW PATIENT'S ATTITUDE*) OR (TI PATIENTS' ATTITUDE* OR AB PATIENTS' ATTITUDE* OR KW PATIENTS' ATTITUDE*) OR (TI Patient Choice* OR AB Patient Choice* OR KW Patient Choice*) OR (TI Patients Choice* OR AB Patients Choice* OR KW Patients Choice*) OR (TI Patient's Choice* OR AB Patient's Choice* OR KW Patient's Choice*) OR (TI Patients' Choice* OR AB Patients' Choice* OR KW Patients' Choice*) OR (TI choice based OR AB choice based OR KW choice based) OR (TI choice-based OR AB choice-based OR KW choice-based) OR (TI PUBLIC PREFERENCE* OR AB PUBLIC PREFERENCE* OR KW PUBLIC PREFERENCE*) OR (TI HEALTH PRIORIT* OR AB HEALTH PRIORIT* OR KW HEALTH PRIORIT*) OR (DE "MEDICAL decision making" OR DE "PATIENT decision making") OR (DE "ATTITUDES toward health" OR DE "PATIENT satisfaction" OR DE "PUBLIC opinion on vaccination") OR (DE "PATIENTS' attitudes" OR DE "ATTITUDES of the terminally ill" OR DE "PATIENT acceptance of health care") OR DE "DISCRETE choice models" OR DE "CONSUMER preferences")	**540**

**Web of Science (All Databases)**	
**Initial search**	
– Most recent date of search: 15 July 2019	
– Date range of search: 2000 to July 2019	
**Query**	**Items found**
TS=((colonoscop* OR coloscop* OR sigmoidoscop* OR stool test* OR Fecal Occult Blood Test* OR Faecal Occult blood test* OR Faecal immunochemical test* OR Fecal immunochemical test*) OR ((colorectal cancer* OR colon cancer* OR bowel cancer* OR rectal cancer*) AND (screening* OR early detection OR early diagnosis)) OR (bowel cancer screening* OR Colorectal Screening* OR colorectal cancer screening* OR Colorectal Prevention* OR Colorectal cancer prevention* OR bowel cancer testing program* OR CRC screening*)) AND TS=(CONJOINT ANALYS* OR CONJOINT-ANALYS* OR CONJOINT STUDY OR CONJOINT STUDIES OR conjoint measurement* OR conjoint-measurement* OR DISCRETE CHOICE* OR DISCRETE-CHOICE* OR DISCRETE RANK* OR ranking* OR ranking exercise* OR rating* OR rating scale* OR STATED PREFERENCE* OR STATED-PREFERENCE* OR PATIENT PREFERENCE* OR PATIENTS PREFERENCE* OR PATIENT'S PREFERENCE* OR PATIENTS' PREFERENCE* OR PATIENT ATTITUDE* OR PATIENTS ATTITUDE* OR PATIENT'S ATTITUDE* OR PATIENTS' ATTITUDE* OR Patient Choice* OR Patients Choice* OR Patient's Choice* OR Patients' Choice* OR choice based OR choice-based OR PUBLIC PREFERENCE* OR HEALTH PRIORIT*)	**5,727**

PsycINFO (via EBSCO)	
**Initial search**	
– Most recent date of search: 12 July 2019	
– Date range of search: 2000 to July 2019	
**Query**	**Items found**
(((TI colonoscop* OR AB colonoscop* OR KW colonoscop*) OR (TI coloscop* OR AB coloscop* OR KW coloscop*) OR (TI sigmoidoscop* OR AB sigmoidoscop* OR KW sigmoidoscop*) OR (TI stool test* OR AB stool test* OR KW stool test*) OR (TI Fecal Occult Blood Test* OR AB Fecal Occult Blood Test* OR KW Fecal Occult Blood Test*) OR (TI Faecal Occult blood test* OR AB Faecal Occult blood test* OR KW Faecal Occult blood test*) OR (TI Faecal immunochemical test* OR AB Faecal immunochemical test* OR KW Faecal immunochemical test*) OR (TI Fecal immunochemical test* OR AB Fecal immunochemical test* OR KW Fecal immunochemical test*)) OR (((TI colorectal cancer* OR AB colorectal cancer* OR KW colorectal cancer*) OR (TI colon cancer* OR AB colon cancer* OR KW colon cancer*) OR (TI bowel cancer* OR AB bowel cancer* OR KW bowel cancer*) OR (TI rectal cancer* OR AB rectal cancer* OR KW rectal cancer*)) AND ((TI screening* OR AB screening* OR KW screening*) OR (TI early detection OR AB early detection OR KW early detection) OR (TI early diagnosis OR AB early diagnosis OR KW early diagnosis) OR (DE "Health Screening" OR DE "Cancer Screening"))) OR ((TI bowel cancer screening* OR AB bowel cancer screening* OR KW bowel cancer screening*) OR (TI Colorectal Screening* OR AB Colorectal Screening* OR KW Colorectal Screening*) OR (TI colorectal cancer screening* OR AB colorectal cancer screening* OR KW colorectal cancer screening*) OR (TI Colorectal Prevention* OR AB Colorectal Prevention* OR KW Colorectal Prevention*) OR (TI Colorectal cancer prevention* OR AB Colorectal cancer prevention* OR KW Colorectal cancer prevention*) OR (TI bowel cancer testing program* OR AB bowel cancer testing program* OR KW bowel cancer testing program*) OR (TI CRC screening* OR AB CRC screening* OR KW CRC screening*))) AND ((TI CONJOINT ANALYS* OR AB CONJOINT ANALYS* OR KW CONJOINT ANALYS*) OR (TI CONJOINT-ANALYS* OR AB CONJOINT-ANALYS* OR KW CONJOINT-ANALYS*) OR (TI CONJOINT STUDY OR AB CONJOINT STUDY OR KW CONJOINT STUDY) OR (TI CONJOINT STUDIES OR AB CONJOINT STUDIES OR KW CONJOINT STUDIES) OR (TI conjoint measurement* OR AB conjoint measurement* OR KW conjoint measurement*) OR (TI conjoint-measurement* OR AB conjoint-measurement* OR KW conjoint-measurement*) OR (TI DISCRETE CHOICE* OR AB DISCRETE CHOICE* OR KW DISCRETE CHOICE*) OR (TI DISCRETE-CHOICE* OR AB DISCRETE-CHOICE* OR KW DISCRETE-CHOICE*) OR (TI DISCRETE RANK* OR AB DISCRETE RANK* OR KW DISCRETE RANK*) OR (TI ranking* OR AB ranking* OR KW ranking*) OR (TI ranking exercise* OR AB ranking exercise* OR KW ranking exercise*) OR (TI rating* OR AB rating* OR KW rating*) OR (TI rating scale* OR AB rating scale* OR KW rating scale*) OR (TI STATED PREFERENCE* OR AB STATED PREFERENCE* OR KW STATED PREFERENCE*) OR (TI STATED-PREFERENCE* OR AB STATED-PREFERENCE* OR KW STATED-PREFERENCE*) OR (TI PATIENT PREFERENCE* OR AB PATIENT PREFERENCE* OR KW PATIENT PREFERENCE*) OR (TI PATIENTS PREFERENCE* OR AB PATIENTS PREFERENCE* OR KW PATIENTS PREFERENCE*) OR (TI PATIENT'S PREFERENCE* OR AB PATIENT'S PREFERENCE* OR KW PATIENT'S PREFERENCE*) OR (TI PATIENTS' PREFERENCE* OR AB PATIENTS' PREFERENCE* OR KW PATIENTS' PREFERENCE*) OR (TI PATIENT ATTITUDE* OR AB PATIENT ATTITUDE* OR KW PATIENT ATTITUDE*) OR (TI PATIENTS ATTITUDE* OR AB PATIENTS ATTITUDE* OR KW PATIENTS ATTITUDE*) OR (TI PATIENT'S ATTITUDE* OR AB PATIENT'S ATTITUDE* OR KW PATIENT'S ATTITUDE*) OR (TI PATIENTS' ATTITUDE* OR AB PATIENTS' ATTITUDE* OR KW PATIENTS' ATTITUDE*) OR (TI Patient Choice* OR AB Patient Choice* OR KW Patient Choice*) OR (TI Patients Choice* OR AB Patients Choice* OR KW Patients Choice*) OR (TI Patient's Choice* OR AB Patient's Choice* OR KW Patient's Choice*) OR (TI Patients' Choice* OR AB Patients' Choice* OR KW Patients' Choice*) OR (TI choice based OR AB choice based OR KW choice based) OR (TI choice-based OR AB choice-based OR KW choice-based) OR (TI PUBLIC PREFERENCE* OR AB PUBLIC PREFERENCE* OR KW PUBLIC PREFERENCE*) OR (TI HEALTH PRIORIT* OR AB HEALTH PRIORIT* OR KW HEALTH PRIORIT*) OR DE "Choice Behavior" OR (DE "Client Attitudes" OR DE "Client Satisfaction") OR DE "Decision Making")	**274**

LIVIVO	
**Initial search**	
– Most recent date of search: 16 July 2019	
– Date range of search: 2000 to July 2019	
**Query**	**Items found**
((colonoscop* OR coloscop* OR sigmoidoscop* OR stool test* OR Fecal Occult Blood Test* OR Faecal Occult blood test* OR Faecal immunochemical test* OR Fecal immunochemical test*) OR ((colorectal cancer* OR colon cancer* OR bowel cancer* OR rectal cancer*) AND (screening* OR early detection OR early diagnosis)) OR (bowel cancer screening* OR Colorectal Screening* OR colorectal cancer screening* OR Colorectal Prevention* OR Colorectal cancer prevention* OR bowel cancer testing program* OR CRC screening*)) AND (CONJOINT ANALYS* OR CONJOINT STUDY OR CONJOINT STUDIES OR conjoint measurement* OR conjoint-measurement* OR DISCRETE CHOICE* OR DISCRETE-CHOICE* OR DISCRETE RANK* OR ranking* OR ranking exercise* OR rating* OR rating scale* OR STATED PREFERENCE* OR STATED-PREFERENCE* OR PATIENT PREFERENCE* OR PATIENTS PREFERENCE* OR PATIENT'S PREFERENCE* OR PATIENTS' PREFERENCE* OR PATIENT ATTITUDE* OR PATIENTS ATTITUDE* OR PATIENT'S ATTITUDE* OR PATIENTS' ATTITUDE* OR Patient Choice* OR Patients Choice* OR Patient's Choice* OR Patients' Choice* OR choice based OR choice-based OR PUBLIC PREFERENCE* OR HEALTH PRIORIT*)	**3,863**

Updating literature search			
**Databases**	**Date range of search**	**Most recent date of search**	**Items found (n)**
PubMed	16.07.2019 to 10.09.2020	10.09.2020	168
	10.09.2020 to 17.11.2021	17.11.2021	194
Embase	16.07.2019 to 10.09.2020	10.09.2020	532
	10.09.2020 to 18.11.2021	18.11.2021	501
Biomedical Reference Collection: Corporate (via EBSCO)	At the time of the update of the systematic literature search, institutional access to the licensed online database Biomedical Reference Collection: Corporate Edition via the Hannover Medical School was no longer available. During our previous literature search, three studies out of the records found in this database met our eligibility criteria and were included in our systematic review. However, all three studies were published between 2000 and 2003 and were also found in at least one of the other bibliographic databases searched. We therefore assume that it is unlikely that we missed any other relevant studies by not being able to rerun our search within this database.		
Web of Science (All Databases)	01/2019 to 10.09.2020	10.09.2020	846
	10.09.2020 to 17.11.2021	17.11.2021	711
PsycINFO (via EBSCO)	07/2019 to 11.09.2020	11.09.2020	10
	09/2020 to 18.11.2021	18.11.2021	9
LIVIVO	01/2019 to 10.09.2020	10.09.2020	486
	09/2020 to 18.11.2021	18.11.2021	865
