# Supplementary material for: Attributes in stated preference elicitation studies on colorectal cancer screening and their relative importance for decision-making among screenees: a systematic review
Source: Health Econ Rev. 2022 Sep 22;12:49. doi: 10.1186/s13561-022-00394-8 (PMC9494881; doi:10.1186/s13561-022-00394-8)
Supplement: Supplementary file 3 — Additional file 3. Risk of bias subdomains and signalling questions based on the approach addressing the certainty of evidence in the relative importance of outcomes or values and preferences developed by GRADE, adapted to conjoint analyses and discrete choice experiments [file 13561_2022_394_MOESM3_ESM.pdf]

Additional file 3 Risk of bias subdomains and signalling questions based on the approach addressing the certainty of evidence in the relative importance of outcomes or values and preferences developed by GRADE, adapted to conjoint analyses and discrete choice experiments

The content is from GRADE (Zhang Y, Alonso-Coello P, Guyatt GH, Yepes-Nuñez JJ, Akl EA, Hazlewood G, et al. GRADE Guidelines: 19. Assessing the certainty of evidence in the importance of outcomes or values and preferences-Risk of bias and indirectness. J Clin Epidemiol. 2019; doi:10.1016/j.jclinepi.2018.01.013).

The adaptations made are listed in the column "Adaptations to CA and DCE" or highlighted in green in the other columns.

Subdomain	Signalling questions	Guidance to answer the question	Decision rule	Adaptation to CA and DCE[a]
Subdomain 1: Selection of participants into the study	Was an appropriate study sample selected from the sampling frame?<br><br>*Answer options[b]*<br>• *Yes: random/probability sample*<br>• *Probably yes: random/probability sample, but only a subset of the target population were likely selected*<br>• *Probably no: opt-in panel representative for target population or random/probability sample of opt-in panel*<br>• *No: non-probability/random sample*	Users should consider:<br>– *The study's sampling strategy (i.e., random sample or consecutive sample, convenience sample, etc.)*, in particular whether only a subset of the target population were likely selected, and if so whether that subset would lead to biased estimates compared to the entire target population.<br><br>**Example** (cross-sectional study):<br>A stratified random sampling strategy would minimize the risk of selecting a study population that is not representative of the sampling frame, while a convenience sample might probably be a biased sample for the study population.<br><br>"In another systematic review to assess the patient preferences for type 2 diabetes treatment-related outcomes, of all 61 included studies, only six showed that the respondents were similar to nonrespondents [47]. Thus, we downgraded the certainty of evidence due to risk of bias resulting from selection of participants into the studies."	When there is only one signalling question for a domain, one study will be classified as low or moderate if the response to the signalling question is yes, or probably yes, and high and critical if the response is no, or probably no	---<br><br>Required to answer "Yes"<br>✓ (Stratified) random sampling<br>✓ Not only a subset of the target population was likely selected

Subdomain	Signalling questions	Guidance to answer the question	Decision rule	Adaptation to CA and DCE[a]
Subdomain 2: Completeness of data	Was the attrition sufficiently low to minimize risk of bias?<br><br>*Answer options*<br>• *Yes*<br>• *Probably yes*<br>• *Probably no*<br>• *No*	Users should consider:<br>– *The response rate.*<br>– *If follow-up was involved, also the attrition rate.*<br>– *The characteristics of the participants who responded and those who did not*.<br><br>This subdomain about missing data includes both the response rate of the study population approached and attrition rate during the follow-up process.<br>High response rates and/or low proportion of loss to follow up are clearly preferable, and a high proportion of nonresponse or dropout could be problematic.<br>Response rate for 80% or higher would be considered high for a cross-sectional study.<br><br>**Example:**<br>To conduct a decision analysis, investigators invited 180 people meeting study eligibility criteria to derive utility measures for health states. Only 64 of the invitees agreed, of whom 57 completed the study. The low response rate is likely to bias the estimates of utilities and impact the credibility of this and future decision analysis based on these results. The answer to the question was no. We classified the study at serious risk of bias in the missing data subdomain.	When there is only one signalling question for a domain, one study will be classified as low or moderate if the response to the signalling question is yes, or probably yes, and high and critical if the response is no, or probably no.<br>The judgement is subjective, users should report transparently the reason for their risk of bias assessment.	– If data on the characteristics of those respondents who did not complete the survey in whole or in part are available, it is important to examine the differences between responders and non-responders or between other subsets of the respondent sample.[1]<br><br>Required to answer "Yes"<br>✓ Response rate of 80% or higher<br>✓ If response rate is less than 80%: Examination of differences between characteristics of responders and non-responders (non-responder analysis – age, sex, and education, income or occupation)<br>✓ If follow-up was involved: Attrition

Subdomain	Signalling questions	Guidance to answer the question	Decision rule	Adaptation to CA and DCE[a]
Subdomain 3: Measurement instrument	1. Was the instrument used for eliciting relative importance of outcomes valid and reliable?<br><br>*Answer options*<br>• *Yes*<br>• *Probably yes*<br>• *Probably no*<br>• *No*	Users should consider:<br>– *Have authors provide information regarding the measurement properties of the instrument they have chosen?*<br>– *Have authors chosen an instrument with which assessors are familiar and with widely accepted reliability and validity?*<br><br>A list of generic instruments with accepted validity and reliability include standard gamble, time trade off, visual analogue scale (or feeling thermometers), discrete choice, treatment trade-off, and willingness to pay. Use of these instruments does not, however, guarantee that they have been administered appropriately.<br><br>**Example:**<br>Polonsky et al. reported a study examining patient preferences regarding a once-weekly glucose-lowering medication. Patient reported their preferences on a 5-point-Likert-type scale ranging from 1 to 5. Given absence of demonstrated validity and reliability for this preference elicitation, our response to the signalling question was "probably not" and we classified this study as serious risk of bias for the subdomain of measurement error. We did not rate it critical risk of bias because five point Likert type scales have proved valid in other contexts.	Please consider yes or probably yes for instruments with accepted validity and reliability.<br><br>If authors have neither used an instrument with widely accepted satisfactory measurement properties, nor have provided information regarding satisfactory reliability and validity, the risk of bias is likely to be substantial.<br><br>To ensure a consistent and objective decision about risk of bias, the risk of bias across a subdomain with more than one signalling questions will be judged with the "lowest response option" that was given to at least one signalling question of the respective subdomain.[b]	---<br><br>Required to answer "Yes"<br>✓ Instrument with accepted validity and reliability (standard gamble, time trade off, visual analogue scale (or feeling thermometers), discrete choice, treatment trade-off, and willingness to pay) was used<br>✓ If an instrument without accepted validity and reliability was used: authors provide information regarding the measurement properties of the instrument they have chosen

Subdomain	Signalling questions	Guidance to answer the question	Decision rule	Adaptation to CA and DCE[a]
	2. Was the instrument administered in the intended way?<br><br>*Answer options*<br>• *Yes*<br>• *Probably yes*<br>• *Probably no*<br>• *No*	Users should consider:<br>– *Have authors demonstrated that the instrument has been administered correctly, or in a manner conforming to their rationale to minimize the risk of introducing bias?*<br>– *Have authors demonstrated that the measurement instruments were administered in a consistent manner across participants and subpopulations?*<br><br>**Example:**<br>Empirically, systematic reviews suggested that the way researchers ask the time trade off questions to elicit preferences my influence the results; this is also true for the standard gamble. In a study assessing the utility of people with traumatic spinal cord injuries, the researchers used a telephone interview strategy to administer the standard gamble. In this study, the participants were asked to dedicate 30-49 minutes to a telephone interview. During the telephone interview, unlike the usual case for standard gamble in which the instrument administration includes a visual prompt, the participants were asked to imagine "that they would live in their current health states for an average life expectancy of 25 years," Additionally, the alternate probabilities in the standard gamble process were only verbally described. For this study, the answer to the question is "no"; we classified this as "serious risk of bias" because the measurement instrument was not administered in the intended way.	To ensure a consistent and objective decision about risk of bias, the risk of bias across a subdomain with more than one signalling questions will be judged with the "lowest response option" that was given to at least one signalling question of the respective subdomain.[b]	– Was mode of administration justified and appropriate (e.g., face-to-face, pen-and-paper, web-based)?[1]<br>– Conjoint-analysis surveys can be administered in many ways (mail surveys using paper-and-pencil, non-mediated paper-and-pencil surveys completed at a finite set of study sites, electronic administration at a finite set of study sites using a laptop computer, or electronic administration via internet).[1]<br>– Use of telephone-based date collection should be precluded, unless the survey instrument is mailed to respondents in advance.[1]<br>– Interviewer-led administration may improve quality of data, because interviewer can recognize that more explanation is needed, can more fully explain the task, and can answer questions.[1]<br><br>Required to answer "Yes"<br>✓ Instrument has been administered face-to-face, pen-and-paper, web-based<br>✓ Consistent administration across participants and subpopulations

Subdomain	Signalling questions	Guidance to answer the question	Decision rule	Adaptation to CA and DCE[a]
	3. Was a valid representation of the outcome (health state) utilized?<br><br>*Answer options*<br>• *Yes*<br>• *Probably yes*<br>• *Probably no*<br>• *No*	Users should consider:<br>– *Optimal representation of the outcome includes a detailed explanation of how the outcome that defines the experience, probability, duration and consequences was developed*.<br><br>**Example:**<br>In the study examining patient preferences regarding a once-weekly glucose-lowering medication option, the researchers presented seven potential outcome characteristics to participants, with five positive and two negative outcomes. The researchers did not report their reason for selecting these outcomes. Moreover, the descriptions of outcomes lacked detail; for example, "once-a-week medication could improve my quality of life". Thus, the answer to this question is "no" because the vague descriptions likely led to varied understanding of the outcome leading us to classify the subdomain as serious risk of bias.	Users should classify study as serious or critical risk of bias only if they have serious doubt regarding the appropriateness of the outcome presentation.<br><br>If the researchers demonstrated they were using available evidence to support the health state presentation, the answer should be yes or probably yes.<br><br>This question only applies when the participants are asked to indicate the importance they would like to place on a set of hypothetical or described outcomes, rather than their own health.<br><br>To ensure a consistent and objective decision about risk of bias, the risk of bias across a subdomain with more than one signalling questions will be judged with the "lowest response option" that was given to at least one signalling question of the respective subdomain.[b]	– Was attribute identification supported by evidence (literature reviews, focus groups, or other scientific methods)?[1]<br>– Was attribute selection justified and consistent with theory (discussion with experts, further pilot testing with subjects, rating/ranking exercises to assess importance of attributes and to facilitate construction of final list of attributes to be included)?[1]<br>– Was level selection for each attribute justified by the evidence and consistent with the study perspective and hypothesis?[1]<br>– Was there sufficient explanation of conjoint tasks?[1]<br>– It is good research practice to introduce attributes and levels prior to the introduction of the actual tasks. It is important to describe all attributes and levels thoroughly and consistently, to ensure that all respondents are evaluating the same task and not making unobservable assumptions about the attributes and levels in a given profile.[1]<br><br>Required to answer "Yes"<br>✓ Attribute identification was supported by evidence<br>✓ Attribute selection was justified and consistent with theory<br>✓ Level selection was supported and justified by evidence and consistent with study perspective and hypothesis<br>✓ Sufficient explanation of choice tasks

Subdomain	Signalling questions	Guidance to answer the question	Decision rule	Adaptation to CA and DCE[a]
	4. Did the researchers check the understanding of the instrument?<br><br>*Answer options*[c]<br>• *Yes:* Understanding was checked by face-to-face pretest interviews AND internal validity of data was assessed and evaluated by internal validity tests<br>• *Probably yes:* Understanding was checked by face-to-face pretest interviews OR internal validity of data was assessed and evaluated by internal validity tests<br>• *Probably no:* Understanding was not checked by face-to-face pretest interviews AND internal validity of data was not assessed and evaluated by internal validity tests BUT no evidence suggesting inadequate understanding<br>• *No:* Understanding was not checked by face-to-face pretest interviews AND internal validity of data was not assessed and evaluated by internal validity tests AND evidence suggesting inadequate understanding	Users should consider:<br>– *Was the instrument simple enough to assume understanding?*<br>– *Did the researchers pilot the instrument?*<br>– *Did the researchers formally test the understanding and did the results suggest understanding of the tasks?*<br><br>Evaluating the risk of bias requires checking whether the study authors have provided evidence of adequate understanding.<br>Checking understanding of participants is, however, neither common practice in the execution nor in the reporting of a study.<br>Fortunately, reviewers may be able to deduce that understanding was adequate if the instrument applied was simple enough, or if the authors describe successful piloting of the instrument.<br><br>**Example:**<br>Gage et al. reported decision and cost-effectiveness analyses comparing warfarin versus aspirin for prophylaxis of stroke. The researchers used the time trade-off technique to elicit the utility of outcomes. Of 69 participants who completed the study, 57 reportedly understood the technique. Nearly 20% of the participants had difficulties with understanding the instrument providing the rationale to classify this study as serious risk of bias in subdomain of measurement instrument.	If the methodology is simple, choosing "The investigators did not formally test the understanding, but the results suggested it was adequate" could be appropriate.<br>If the researches piloted the methodology, choosing "The investigator did not formally test the understanding, but there was evidence suggesting adequate understanding" may also be appropriate.<br><br>To ensure a consistent and objective decision about risk of bias, the risk of bias across a subdomain with more than one signalling questions will be judged with the "lowest response option" that was given to at least one signalling question of the respective subdomain.[b]	– It is important to include face-to-face pretest interviews and a quantitative pilot test as part of the construction of the data collection instrument. Careful pretesting can identify areas of misunderstanding or common errors, as well as whether the survey is too long. In addition, it can reveal whether respondents understand the instructions and feel the questions are appropriate.[1]<br>– The quality of responses can be assessed by evaluating the internal validity of the data. Examples include a repeated question, an alternative whose attribute levels are all better than the attributes of another alternative in the choice set, or three questions that support a check of preference transitivity[1]<br>– Iterative face-to-face pilot testing is needed to guide development and testing of DCE surveys. This includes testing respondent understanding of choice contexts, generation and testing of appropriateness and understanding of attributes/levels, task complexity, length, timing and likely response rates.[2]<br>– Validity refers to the degree to which a study succeeds in measuring the intended values by overcoming potential biases and the hypothetical nature of the valuation exercise. It is customary to include built-in tests in DCEs in order to check if the standard assumptions (axioms) of utility theory hold, for example, rationality (internal consistency) of responses, dominant choice sets, transitivity and monotonicity[3]<br><br>Required to answer "Yes"<br>✓ Understanding was checked by face-to-face pretest interviews<br>✓ Internal validity of the data was assessed and evaluated by internal validity tests

Subdomain	Signalling questions	Guidance to answer the question	Decision rule	Adaptation to CA and DCE[a]
Subdomain 4: Data analysis	Where the results analysed appropriately to avoid influence of bias and confounding?<br><br>*Answer options*<br>• *Yes*<br>• *Probably yes*<br>• *Probably no*<br>• *No*	Users should consider:<br>– *Was the adjustment, stratification, or model selection appropriate?* Studies addressing the importance of outcomes should present results adjusted for important co-variates. For example, if the relative importance of an outcome is associated with participants' age and this can be appropriately controlled for, reporting adjusted results is likely to be informative.<br><br>For some methodologies, such as discrete choice experiments, researchers need to select appropriate models and adjust potential characteristics that could distort the results and conclusion.<br>Raters need to take data analysis into the risk of bias consideration, with scrutiny on the adjustment, stratifications, model selections and interactions.*<br>This domain may not be applicable to all primary studies because not all studies will require controlled data analysis.<br><br>**Example:**<br>In a discrete choice exercise study, the researchers invited 489 screening-naïve and 496 screened individuals to determine preferences of various screening tests and to predict uptake for colorectal cancer screening programs. The researchers applied multi-nominal logit model to analyse the data. Although they conducted sensitivity analyses to include irrational responses, they did not conduct other sensitivity analyses. And although they reported respondent characteristics, they did not examine the interaction between choice and respondent characteristics and only compared differences among subgroups with Chi-square and Student's t-Tests. Thus, our answer to the signalling question was probably not, and we classified this study as serious risk of bias for data analysis.	When there is only one signalling question for a domain, one study will be classified as low or moderate if the response to the signalling question is yes, or probably yes, and high and critical if the response is no, or probably no.	– Preference variation among individuals that is unaccounted for in modeling can result in biased estimates. Variations in preferences that arise from differences in individual characteristics such as age, education, gender, and health status can be incorporated into the modeling process by interacting individual characteristics with the attributes included in the conjoint analysis.[1]<br>– Researchers also may consider split-sample analysis if sample sizes are sufficiently large.[1]<br><br>Required to answer "Yes"<br>✓ Stratification<br>OR<br>✓ Interactions between individual characteristics and attributes included in the choice tasks were analysed<br>OR<br>✓ Latent-class analysis (segmentation as an alternative to stratification)

CA=conjoint analysis; DCE=discrete choice experiment
[a]Additional information extracted out of literature discussing the process of carrying out a choice study to generate guidance and criteria required for answering the signalling questions with "Yes" in the context of CAs and DCEs; [b]Adapted; [c]Modification of original answer options

[1] Bridges JFP, Hauber AB, Marshall D, Lloyd A, Prosser LA, Regier DA, et al. Conjoint analysis applications in health-a checklist: a report of the ISPOR Good Research Practices for Conjoint Analysis Task Force. Value Health. 2011;14:403–13. doi:10.1016/j.jval.2010.11.013.; [2] Lancsar E, Louviere J. Conducting discrete choice experiments to inform healthcare decision making. A user´s guide. Pharmacoeconomics. 2008;26:661–77.; [3] Ryan M, Gerard K, Amaya-Amaya M. Using discrete choice experiments to value health and health care. Dordrecht: Springer; 2008.
