# Supplementary material for: Attributes in stated preference elicitation studies on colorectal cancer screening and their relative importance for decision-making among screenees: a systematic review
Source: Health Econ Rev. 2022 Sep 22;12:49. doi: 10.1186/s13561-022-00394-8 (PMC9494881; doi:10.1186/s13561-022-00394-8)
Supplement: Supplementary file 4 — Additional file 4. List of records excluded due to full-text screening and primary reasons for exclusion [file 13561_2022_394_MOESM4_ESM.pdf]

Attributes in stated preference elicitation studies on colorectal cancer screening and their relative importance for decision-making among screenees: a systematic review

Additional file 4 List of records excluded due to full-text screening and primary reasons for exclusion

Reference	Primary reason for exclusion
Almog R, Ezra G, Lavi I, Rennert G, Hagoel L. The public prefers fecal occult blood test over colonoscopy for colorectal cancer screening. Eur J Cancer Prev. 2008;17:430–7.	No conjoint analysis or discrete choice experiment
Angtuaco TL, Banaad-Omiotek GD, Howden CW. Differing attitudes toward virtual and conventional colonoscopy for colorectal cancer screening: surveys among primary care physicians and potential patients. Am J Gastroenterol. 2001;96:887–93.	No conjoint analysis or discrete choice experiment
Banks J, Hollinghurst S, Bigwood L, Peters TJ, Walter FM, Hamilton W. Preferences for cancer investigation: a vignette-based study of primary-care attendees. Lancet Oncol. 2014;15:232–40.	No conjoint analysis or discrete choice experiment
Bender D., Imaeda A.B., Fraenkel L. Patient preferences for colon cancer screening tests. Gastrointest Endosc. 2009;69:AB312.	Conference abstract only
Bonello B, Ghanouni A, Bowyer HL, MacRae E, Atkin W, Halloran SP, et al. Using a hypothetical scenario to assess public preferences for colorectal surveillance following screening-detected, intermediate-risk adenomas: annual home-based stool test vs. triennial colonoscopy. BMC Gastroenterol. 2016;16:113.	No conjoint analysis or discrete choice experiment
Boone D, Mallett S, Zhu S, Yao GL, Bell N, Ghanouni A, et al. Patients' & healthcare professionals' values regarding true- & false-positive diagnosis when colorectal cancer screening by CT colonography: discrete choice experiment. PLoS One. 2013;8:e80767.	CT colonography only
Calderwood AH, Wasan SK, Heeren TC, Schroy PC. Patient and provider preferences for colorectal cancer screening: How does ct colonography compare to other modalities? Int J Canc Prev. 2011;4:307–38.	No conjoint analysis or discrete choice experiment
Chablani SV, Cohen N, White D, Itzkowitz SH, DuHamel K, Jandorf L. Colorectal Cancer Screening Preferences among Black and Latino Primary Care Patients. J Immigr Minor Health. 2017;19:1100–8.	No conjoint analysis or discrete choice experiment
Chambers JA, Callander AS, Grangeret R, O'Carroll RE. Attitudes towards the Faecal Occult Blood Test (FOBT) versus the Faecal Immunochemical Test (FIT) for colorectal cancer screening: perceived ease of completion and disgust. BMC Cancer. 2016;16:96.	No conjoint analysis or discrete choice experiment
Chatrath H, Rex DK. Potential screening benefit of a colorectal imaging capsule that does not require bowel preparation. J Clin Gastroenterol. 2014;48:52–4.	No conjoint analysis or discrete choice experiment
Cheng J, Pullenayegum E, Marshall DA, Marshall JK, Thabane L. An empirical comparison of methods for analyzing correlated data from a discrete choice survey to elicit patient preference for colorectal cancer screening. BMC Med Res Methodol. 2012;15.	Duplicate
Cho Y-H, Kim DH, Cha JM, Jeen YT, Moon JS, Kim J-O, et al. Patients' Preferences for Primary Colorectal Cancer Screening: A Survey of the National Colorectal Cancer Screening Program in Korea. Gut Liver. 2017;11:821–7.	No conjoint analysis or discrete choice experiment
Choi KS, Kwak M-S, Lee H-Y, Jun JK, Hahm M-I, Park E-C. Screening for Gastric Cancer in Korea: Population-Based Preferences for Endoscopy versus Upper Gastrointestinal Series. Cancer Epidemiol Biomarkers Prev. 2009;18:1390–8.	Methods for gastric cancer screening only
Dolan JG. Patient priorities in colorectal cancer screening decisions. Health Expect. 2005;8:334–44.	No conjoint analysis or discrete choice experiment
Dolan JG, Boohaker E, Allison J, Imperiale TF. Patients' preferences and priorities regarding colorectal cancer screening. Med Decis Making. 2013;33:59–70.	No conjoint analysis or discrete choice experiment
Dolan JG, Boohaker E, Allison J, Imperiale TF. Can Streamlined Multicriteria Decision Analysis Be Used to Implement Shared Decision Making for Colorectal Cancer Screening? Med Decis Making. 2014;34:746–55.	No conjoint analysis or discrete choice experiment
Frew EJ, Wolstenholme JL, Whynes DK. Eliciting relative preferences for two methods of colorectal cancer screening. Eur J Cancer Care (Engl). 2005;14:124–31.	No conjoint analysis or discrete choice experiment
Fritzell K, Nilsson KS, Jervaeus A, Hultcrantz R, Wengstrom Y. The importance of people's values and preferences for colorectal cancer screening participation. Eur J Public Health. 2017;27:1079–84.	No conjoint analysis or discrete choice experiment

Reference	Primary reason for exclusion
Gareen IF, Siewert B, Vanness DJ, Herman B, Johnson CD, Gatsonis C. Patient willingness for repeat screening and preference for CT colonography and optical colonoscopy in ACRIN 6664: the National CT Colonography trial. Patient Prefer Adherence. 2015;9:1043–51.	No conjoint analysis or discrete choice experiment
Ghanouni A, Halligan S, Taylor SA, Boone D, Plumb A, Stoffel S, et al. Quantifying public preferences for different bowel preparation options prior to screening CT colonography: a discrete choice experiment. BMJ Open. 2014;4:e004327.	Bowel preparation options only
Griffith JM, Fichter M, Fowler FJ, Lewis C, Pignone MP. Should a colon cancer screening decision aid include the option of no testing? A comparative trial of two decision aids. BMC Med Inform Decis Mak. 2008;8:10.	No conjoint analysis or discrete choice experiment
Griffith JM, Lewis CL, Brenner ART, Pignone MP. The effect of offering different numbers of colorectal cancer screening test options in a decision aid: a pilot randomized trial. BMC Med Inform Decis Mak. 2008;8:4.	No conjoint analysis or discrete choice experiment
Hawley S, Lillie S, Cooper G, Lafata JE. Managed care patients' preferences, physician recommendations, and colon cancer screening. Am J Manag Care. 2014;20:555–61.	No conjoint analysis or discrete choice experiment
Hawley ST, McQueen A, Bartholomew LK, Greisinger AJ, Coan SP, Myers R, Vernon SW. Preferences for colorectal cancer screening tests and screening test use in a large multispecialty primary care practice. Cancer. 2012;118:2726–34.	No conjoint analysis or discrete choice experiment
Ho W, Broughton DE, Donelan K, Gazelle GS, Hur C. Analysis of barriers to and patients' preferences for CT colonography for colorectal cancer screening in a nonadherent urban population. AJR Am J Roentgenol. 2010;195:393–7.	CT colonography only
Hoffman RM, Elmore JG, Pignone MP, Gerstein BS, Levin CA, Fairfield KM. Knowledge and values for cancer screening decisions: Results from a national survey. Patient Educ Couns. 2016;99:624–30.	No conjoint analysis or discrete choice experiment
Hollinghurst S, Banks J, Bigwood L, Walter FM, Hamilton W, Peters TJ. Using willingness-to-pay to establish patient preferences for cancer testing in primary care. BMC Med Inform Decis Mak. 2016;16:105.	No conjoint analysis or discrete choice experiment
Howard K, Salkeld G, Pignone M, Hewett P, Cheung P, Olsen J, et al. Preferences for CT colonography and colonoscopy as diagnostic tests for colorectal cancer: a discrete choice experiment. Value Health. 2011;14:1146–52.	Participants with indications for diagnostic colonoscopy only
Hummel JM, Steuten LGM, Groothuis-Oudshoorn CJM, Mulder N, Ijzerman MJ. Preferences for colorectal cancer screening techniques and intention to attend: a multi-criteria decision analysis. Appl Health Econ Health Policy. 2013;11:499–507.	No conjoint analysis or discrete choice experiment
Imaeda A, Bender D, Fraenkel L. What is most important to patients when deciding about colorectal screening? J Gen Intern Med. 2010;25:688–93.	No conjoint analysis or discrete choice experiment
Janssen EM, Pollack CE, Boyd C, Bridges JFP, Xue Q-L, Wolff AC, Schoenborn NL. How Do Older Adults Consider Age, Life Expectancy, Quality of Life, and Physician Recommendation When Making Cancer Screening Decisions? Results from a National Survey Using a Discrete Choice Experiment. Med Decis Making. 2019:272989X19853516.	No colorectal cancer screening test related attributes or levels
Janz NK, Lakhani I, Vijan S, Hawley ST, Chung LK, Katz SJ. Determinants of colorectal cancer screening use, attempts, and non-use. Prev Med. 2007;44:452–8.	No conjoint analysis or discrete choice experiment
Jones RM, Woolf SH, Cunningham TD, Johnson RE, Krist AH, Rothemich SF, Vernon SW. The relative importance of patient-reported barriers to colorectal cancer screening. Am J Prev Med. 2010;38:499–507.	No conjoint analysis or discrete choice experiment
Jung HS, Park DK, Kim MJ, Yu SK, Kwon KA, Ku YS, et al. A comparison of patient acceptance and preferences between CT colonography and conventional colonoscopy in colorectal cancer screening. Korean J Intern Med. 2009;24:43–7.	No conjoint analysis or discrete choice experiment
Katsumura Y, Yasunaga H, Imamura T, Ohe K, Oyama H. Relationship between risk information on total colonoscopy and patient preferences for colorectal cancer screening options: analysis using the analytic hierarchy process. BMC Health Serv Res. 2008;8:106.	No conjoint analysis or discrete choice experiment
Kilambi V, Johnson FR, Gonzalez JM, Mohamed AF. Valuations of genetic test information for treatable conditions: the case of colorectal cancer screening. Value Health. 2014;17:838–45.	Genetic testing alternatives only

Reference	Primary reason for exclusion
Kistler CE, Hess TM, Howard K, Pignone MP, Crutchfield TM, Hawley ST, et al. Older adults' preferences for colorectal cancer-screening test attributes and test choice. Patient Prefer Adherence. 2015;9:1005-1016.	Duplicate
Lahat A, Assouline-Dayan Y, Katz LH, Fidder HH. The preference for an endoscopist specific sex: a link between ethnic origin, religious belief, socioeconomic status, and procedure type. Patient Prefer Adherence. 2013;7:897–903.	No conjoint analysis or discrete choice experiment
Le Pimpec F, Moutel G, Piette C, Lievre A, Bretagne J-F. Fecal immunological blood test is more appealing than the guaiac-based test for colorectal cancer screening. Dig Liver Dis. 2017;49:1267–72.	No conjoint analysis or discrete choice experiment
Lee JM, Kim ES, Chun HJ, Yoo IK, Lee JM, Kim SH, et al. Is There a Change in Patient Preference for a Female Colonoscopist during the Last Decade in Korea? Clin Endosc. 2018;51:72–9.	No conjoint analysis or discrete choice experiment
Lewis Carmen, Fowler Floyd J, Fichter Marlie, Griffith Jennifer M, Pignone Michael P. Should a colon cancer screening decision aid include the option of no testing? A comparative trial of two decision aids. BMC Med Inform Decis Mak. 2008;8.	Duplicate
Ling BS, Moskowitz MA, Wachs D, Pearson B, Schroy PC. Attitudes toward colorectal cancer screening tests. J Gen Intern Med. 2001;16:822–30.	No conjoint analysis or discrete choice experiment
Marbet UA, Bauerfeind P, Brunner J, Dorta G, Valloton JJ, Delco F. Colonoscopy is the preferred colorectal cancer screening method in a population-based program. Endoscopy. 2008;40:650–5.	No conjoint analysis or discrete choice experiment
Marshall DA, Johnson FR, Kulin NA, Ozdemir S, Walsh JME, Marshall JK, et al. How do physician assessments of patient preferences for colorectal cancer screening tests differ from actual preferences? A comparison in Canada and the United States using a stated-choice survey. Health Econ. 2009;18:1420–39.	Duplicate
McEntire J, Sahota J, Hydes T, Trebble TM. An evaluation of patient attitudes to colonoscopy and the importance of endoscopist interaction and the endoscopy environment to satisfaction and value. Scand J Gastroenterol. 2013;48:366–73.	No conjoint analysis or discrete choice experiment
Menees SB, Inadomi JM, Korsnes S, Elta GH. Women patients' preference for women physicians is a barrier to colon cancer screening. Gastrointest Endosc. 2005;62:219–23.	No conjoint analysis or discrete choice experiment
Messina CR, Lane DS, Grimson R. Colorectal cancer screening attitudes and practices - Preferences for decision making. Am J Prev Med. 2005;28:439–46.	No conjoint analysis or discrete choice experiment
Miles A, Rodrigues V, Sevdalis N. The effect of information about false negative and false positive rates on people's attitudes towards colorectal cancer screening using faecal occult blood testing (FOBt). Patient Educ Couns. 2013;93:342–9.	No conjoint analysis or discrete choice experiment
Moawad FJ, Maydonovitch CL, Cullen PA, Barlow DS, Jenson DW, Cash BD. CT colonography may improve colorectal cancer screening compliance. AJR Am J Roentgenol. 2010;195:1118–23.	No conjoint analysis or discrete choice experiment
Mohd Suan MA, Mohammed NS, Abu Hassan MR. Colorectal Cancer Awareness and Screening Preference: A Survey during the Malaysian World Digestive Day Campaign. Asian Pac J Cancer Prev. 2015;16:8345–9.	No conjoint analysis or discrete choice experiment
Moreno CC, Jarrett T, Vey BL, Mittal PK, Krupinski EA, Roberts DL. Patient Knowledge Regarding Colorectal Cancer Risk, Opinion of Screening, and Preferences for a Screening Test. Curr Probl Diagn Radiol. 2019;48:50–2.	No conjoint analysis or discrete choice experiment
Moreno CC, Weiss PS, Jarrett TL, Roberts DL, Mittal PK, Votaw JR. Patient Preferences Regarding Colorectal Cancer Screening: Test Features and Cost Willing to Pay Out of Pocket. Curr Probl Diagn Radiol. 2016;45:189–92.	No conjoint analysis or discrete choice experiment
Nelson RL, Schwartz A. A survey of individual preference for colorectal cancer screening technique. BMC Cancer. 2004;4.	No conjoint analysis or discrete choice experiment
Nelson RL, Schwartz A, Pavel D. Assessment of the usefulness of a diagnostic test: a survey of patient preference for diagnostic techniques in the evaluation of intestinal inflammation. BMC Med Res Methodol. 2001;1.	No conjoint analysis or discrete choice experiment
Ohe K, Imamura T, Yasunaga H, Katsumura Y, Oyama H. Relationship between risk information on total colonoscopy and patient preferences for colorectal cancer screening options: Analysis using the Analytic Hierarchy Process. BMC Health Serv Res. 2008;8.	Duplicate

Reference	Primary reason for exclusion
Osborne JM, Wilson C, Moore V, Gregory T, Flight I, Young GP. Sample preference for colorectal cancer screening tests: Blood or stool? Open Journal of Preventive Medicine. 2012;2:326–31.	No conjoint analysis or discrete choice experiment
Pham R, Cross S, Fernandez B, Corson K, Dillon K, Yackley C, Davis MM. "Finding the Right FIT": Rural Patient Preferences for Fecal Immunochemical Test (FIT) Characteristics. J Am Board Fam Med. 2017;30:632–44.	No conjoint analysis or discrete choice experiment
Powell AA, Burgess DJ, Vernon SW, Griffin JM, Grill JP, Noorbaloochi S, Partin MR. Colorectal cancer screening mode preferences among US veterans. Prev Med. 2009;49:442–8.	No conjoint analysis or discrete choice experiment
Priaulx J, Csanadi M, Koning HJd, McKee M. A choice experiment to identify the most important elements of a successful cancer screening program according to those who research and manage such programs. Int J Health Plann Manage. 2019;34:e34-e45.	Cancer screening experts as target population only
Rex DK, Lieberman DA. A survey of potential adherence to capsule colonoscopy in patients who have accepted or declined conventional colonoscopy. J Clin Gastroenterol. 2012;46:691–5.	No conjoint analysis or discrete choice experiment
Rogers CR, Goodson P, Dietz LR, Okuyemi KS. Predictors of Intention to Obtain Colorectal Cancer Screening Among African American Men in a State Fair Setting. Am J Mens Health. 2018;12:851–62.	No conjoint analysis or discrete choice experiment
Ryan M, San Miguel F. Revisiting the axiom of completeness in health care. Health Econ. 2003;12:295–307.	Students as target population only
Saengow U, Chongsuwiwatvong V, Geater A, Birch S. Preferences and acceptance of colorectal cancer screening in Thailand. Asian Pac J Cancer Prev. 2015;16:2269–76.	No conjoint analysis or discrete choice experiment
Salkeld GP, Solomon MJ, Short L, Ward J. Measuring the importance of attributes that influence consumer attitudes to colorectal cancer screening. ANZ J Surg. 2003;73:128–32.	No conjoint analysis or discrete choice experiment
Salkeld G, Solomon M, Short L, Ryan M, Ward JE. Evidence-based consumer choice: a case study in colorectal cancer screening. Aust N Z J Public Health. 2003;27:449–55.	Duplicate
Sava MG, Dolan JG, May JH, Vargas LG. A Personalized Approach of Patient-Health Care Provider Communication Regarding Colorectal Cancer Screening Options. Med Decis Making. 2018;38:601–13.	No conjoint analysis or discrete choice experiment.
Schroy PC, Lal S, Glick JT, Robinson PA, Zamor P, Heeren TC. Patient preferences for colorectal cancer screening: How does stool DNA testing fare? Am J Manag Care. 2007;13:393–400.	No conjoint analysis or discrete choice experiment
Schroy PC, Glick JT, Robinson PA, Heeren T. Screening preferences of patients at familial risk of colorectal cancer. Dig Dis Sci. 2007;52:2788–95.	No conjoint analysis or discrete choice experiment
Schroy PC, Heeren TC. Patient perceptions of stool-based DNA testing for colorectal cancer screening. Am J Prev Med. 2005;28:208–14.	No conjoint analysis or discrete choice experiment
Schwartz A, Nelson RL. A survey of individual preference for colorectal cancer screening technique. BMC Cancer. 2004;4.	Duplicate
Shah DK, Karasek V, Gerkin RD, Ramirez FC, Young MA. Sex preferences for colonoscopists and GI physicians among patients and health care professionals. Gastrointest Endosc. 2011;74:122-127.e2.	No conjoint analysis or discrete choice experiment
Sheikh RA, Kapre S, Calof OM, Ward C, Raina A. Screening preferences for colorectal cancer: a patient demographic study. South Med J. 2004;97:224–30.	No conjoint analysis or discrete choice experiment
Shokar NK, Carlson CA, Weller SC. Informed decision making changes test preferences for colorectal cancer screening in a diverse population. Ann Fam Med. 2010;8:141–50.	No conjoint analysis or discrete choice experiment
Taber JM, Aspinwall LG, Heichman KA, Kinney AY. Preferences for blood-based colon cancer screening differ by race/ethnicity. Am J Health Behav. 2014;38:351–61.	No conjoint analysis or discrete choice experiment
Van Dam L, Korfage IJ, Kuipers EJ, Hol L, van Roon, AHC, Reijerink, JCIY, et al. What influences the decision to participate in colorectal cancer screening with faecal occult blood testing and sigmoidoscopy? Eur J Cancer. 2013;49:2321–30.	No conjoint analysis or discrete choice experiment
Veldwijk J, Essers BAB, Lambooij MS, Dirksen CD, Smit HA, Wit GA de. Survival or Mortality: Does Risk Attribute Framing Influence Decision-Making Behavior in a Discrete Choice Experiment? Value Health. 2016;19:202–9.	Genetic screening only

Reference	Primary reason for exclusion
Veldwijk J, Groothuis-Oudshoorn CGM, Kihlbom U, Langenskiold S, Dekker E, Kallenberg FGJ, et al. How psychological distance of a study sample in discrete choice experiments affects preference measurement: a colorectal cancer screening case study. Patient Prefer Adherence. 2019;13:273–82.	Genetic screening only
Veldwijk J, Lambooij MS, Kallenberg FGJ, van Kranen HJ, Bredenoord AL, Dekker E, et al. Preferences for genetic testing for colorectal cancer within a population-based screening program: a discrete choice experiment. Eur J Hum Genet. 2016;24:361–6.	Genetic screening only
Weymann D, Veenstra DL, Jarvik GP, Regier DA. Patient preferences for massively parallel sequencing genetic testing of colorectal cancer risk: a discrete choice experiment. Eur J Hum Genet. 2018;26:1257–65.	Genetic screening only
Wolf MS, Rademaker A, Bennett CL, Ferreira MR, Dolan NC, Davis TC, et al. Development of a Brief Survey on Colon Cancer Screening Knowledge and Attitudes Among Veterans. Prev Chronic Dis. 2005;2.	No conjoint analysis or discrete choice experiment
Wolf RL, Basch CE, Brouse CH, Shmukler C, Shea S. Patient preferences and adherence to colorectal cancer screening in an urban population. Am J Public Health. 2006;96:809–11.	No conjoint analysis or discrete choice experiment
Wolf RL, Basch CE, Zybert P, Basch CH, Ullman R, Shmukler C, et al. Patient Test Preference for Colorectal Cancer Screening and Screening Uptake in an Insured Urban Minority Population. J Community Health. 2016;41:502–8.	No conjoint analysis or discrete choice experiment
Wong MCS, Tsoi KKF, Ng SSM, Lou VWQ, Choi SYP, Ling KWK, et al. A comparison of the acceptance of immunochemical faecal occult blood test and colonoscopy in colorectal cancer screening: a prospective study among Chinese. Aliment Pharmacol Ther. 2010;32:74–82.	No conjoint analysis or discrete choice experiment
Xu Y, Levy BT, Daly JM, Bergus GR, Dunkelberg JC. Comparison of patient preferences for fecal immunochemical test or colonoscopy using the analytic hierarchy process. BMC Health Serv Res. 2015;15:175.	No conjoint analysis or discrete choice experiment
Zajac I, Duncan A, Turnbull D, Wilson C, Flight I. Blood-based screening for bowel cancer may not resolve suboptimal screening participation in Australia. Aust N Z J Public Health.	No conjoint analysis or discrete choice experiment
Zajac I, Duncan A, Turnbull D, Wilson C, Flight I. Blood-based screening for bowel cancer may not resolve suboptimal screening participation in Australia. Aust N Z J Public Health. 2016;40:337–41.	Duplicate

CT=computed tomographic

Updating literature search	
**Reference**	**Primary reason for exclusion**
Berchi C, Launoy G. [Participation in organized screening for bowel cancer and screening for prostate cancer]. Sante Publique. 2019;S2(HS2):13-32.	Non-English language
Hyams T, Golden B, Sammarco J, Sultan S, King-Marshall E, Wang MQ, Curbow B. Evaluating preferences for colorectal cancer screening in individuals under age 50 using the Analytic Hierarchy Process. BMC Health Services Research. 2021;21:754.	No conjoint analysis or discrete choice experiment
Labaeka EO, Irabor AE, Irabor DO. Fecal Immunochemical Test as a Screening Method for Colorectal Cancer in University College Hospital Ibadan, Nigeria. JCO Glob Oncol. 2020;6:525–31.	No conjoint analysis or discrete choice experiment
MacLeod C, Wilson P, Watson AJM. Colon capsule endoscopy: an innovative method for detecting colorectal pathology during the COVID-19 pandemic? Colorectal Dis. 2020;22:621–4.	No conjoint analysis or discrete choice experiment
Makaroff KE, Khalil C, Spiegel B, Ahluwalia SC, Almario CV. Insights into patients' knowledge, attitudes, beliefs, and main drivers of decision making when selecting among different colorectal cancer (crc) screening options: Thematic analysis of patient focus groups. Gastroenterology. 2021;160:S-201-S-202.	No conjoint analysis or discrete choice experiment
Rutter MD, Evans R, Hoare Z, Wagner C von, Deane J, Esmaily S, et al. WASh multicentre randomised controlled trial: water-assisted sigmoidoscopy in English NHS bowel scope screening. Gut. 2021;70:845–52.	No conjoint analysis or discrete choice experiment
Sandoval JL, Relecom A, Ducros C, Bulliard J-L, Arzel B, Guessous I. Screening Status as a Determinant of Choice of Colorectal Cancer Screening Method: A Population-Based Informed Survey. Gastrointest Tumors. 2021;8:63–70.	No conjoint analysis or discrete choice experiment
Usher-Smith JA, Mills KM, Riedinger C, Saunders CL, Helsingen LM, Lytvyn L, et al. The impact of information about different absolute benefits and harms on intention to participate in colorectal cancer screening: A think-aloud study and online randomised experiment. PLOS ONE. 2021;16:e0246991.	No conjoint analysis or discrete choice experiment
Wagner C von, Verstraete W, Hirst Y, Nicholson BD, Stoffel ST, Laszlo H. Public preferences for using quantitative faecal immunochemical test versus colonoscopy as diagnostic test for colorectal cancer: evidence from an online survey. BJGP Open. 2020;4:1–13.	No conjoint analysis or discrete choice experiment
Zhu X, Parks PD, Weiser E, Fischer K, Griffin JM, Limburg PJ, Finney Rutten LJ. National Survey of Patient Factors Associated with Colorectal Cancer Screening Preferences. Cancer Prev Res (Phila). 2021;14:603–14.	No conjoint analysis or discrete choice experiment
Zhu X, Weiser E, Jacobson DJ, Griffin JM, Limburg PJ, Finney Rutten LJ. Patient preferences on general health and colorectal cancer screening decision-making: Results from a national survey. Patient Educ Couns 2021.	No conjoint analysis or discrete choice experiment
