# Supplementary material for: Attributes in stated preference elicitation studies on colorectal cancer screening and their relative importance for decision-making among screenees: a systematic review
Source: Health Econ Rev. 2022 Sep 22;12:49. doi: 10.1186/s13561-022-00394-8 (PMC9494881; doi:10.1186/s13561-022-00394-8)
Supplement: Supplementary file 6 — Additional file 6. Calculated relative importance of attributes per study [file 13561_2022_394_MOESM6_ESM.pdf]

Additional file 6 Calculated relative importance of attributes per study

Study	Attributes	RI	Score
Rating			
Hawley et al. 2008	What the test involved	37	4
	Accuracy	19	3
	Frequency	17	5
	Discomfort	15	2
	Preparation	13	1
Ranking			
Gyrd-Hansen et al. 2001	Out of pocket expense	8,67† 7,39‡	2
	Number of screening tests performed over a lifetime	n.s.	n.s.
	Risk of false positive diagnosis over a lifetime	n.s.	n.s.
	Risk reduction over a lifetime	91,33† 92,61‡	1
Discrete Choice – Generic – Frequencies			
Pignone et al. 2012	Ability to reduce CRC incidence and mortality	56	1
	Frequency	20	2
	Nature of test	18	3
	Complications	4	4
	Discomfort	2	5
	Out of pocket costs	0	6
Brenner et al. 2014	Reduction in risk of getting or dying from CRC	49.3	1
	Nature of the test	20.6	2
	Frequency of the test	16.7	3
	Chance of complications	9.8	4
	Chance of needing a colonoscopy over 10 years	3.6	5
Pignone et al. 2014	Follow-up care cost you pay	34	1
	Money paid for screening	33	2
	Testing options	17	3
	Travel time	16	4
Kistler et al. 2015	Testing procedure	35	1
	Mortality reduction	26	2
	Test frequency	19	3
	Risk of complications	19	4
Martens et al. 2016	Follow-up care cost you pay	42	1
	Money paid for screening	28	2
	Testing options	15	3
	Travel time	15	4
Discrete Choice – Generic – Coefficients			
Salkeld et al. 2000	Diet and medication restrictions	22,33	2
	Whether GP supervizes test	15,06	3
	Notification of negative test result	n.s.	n.s.
	Cost of the test kit	60,76	1
	Chance of a false positive	1,84	4
Salkeld et al. 2003	Benefit	82,50	1
	Harm	2,94	3
	Notification	14,56	2
Marshall et al. 2007	Process	11,93	4
	Preparation	12,30	3
	Pain	6,39	5
	Specificity	19,13	2
	Sensitivity	50,25	1
	Cost	*	*

Study	Attributes	RI	Score
Howard et al. 2009<br>Frame 1	Sensitivity - Accuracy of test for cancers (How many cancers the test will find)	47,92	1
	Sensitivity - Accuracy of test for large polyps (How many large polyps the test will find)	16,91	3
	Specificity - How accurate the test is at saying you do not have cancer (The number of people who are correctly reassured by the test that they do not have cancer)	27,66	2
	Cost	7,51	4
	Dietary and medication restrictions	N/A	N/A
	How the sample is collected	N/A	N/A
Frame 2	Sensitivity - Accuracy of test for cancers (How many cancers the test will miss)	58,62	1
	Sensitivity - Accuracy of test for large polyps (How many large polyps the test will miss)	21,68	2
	Specificity - How accurate the test is at saying you do not have cancer (The number of people who have unnecessary colonoscopies)	13,45	3
	Cost	6,25	4
	Dietary and medication restrictions	N/A	N/A
	How the sample is collected	N/A	N/A
Frame 3	Sensitivity - Accuracy of test for cancers (How many cancers the test will find)	61,96	1
	Sensitivity - Accuracy of test for large polyps (How many large polyps the test will find)	25,27	2
	Specificity - How accurate the test is at saying you do not have cancer (The number of people who have unnecessary colonoscopies)	7,00	3
	Cost	5,77	4
	Dietary and medication restrictions	N/A	N/A
	How the sample is collected	N/A	N/A
Frame 4	Sensitivity - Accuracy of test for cancers (How many cancers the test will miss)	54,28	1
	Sensitivity - Accuracy of test for large polyps (How many large polyps the test will miss)	23,24	2
	Specificity - How accurate the test is at saying you do not have cancer (The number of people who are correctly reassured by the test that they do not have cancer)	10,52	4
	Cost	11,96	3
	Dietary and medication restrictions	N/A	N/A
	How the sample is collected	N/A	N/A
Marshall et al. 2009			
Canadian	Process	15,28	4
	Frequency	6,84	7
	Follow-up	n.s.	n.s.
	Pain	16,14	3
	Preparation	8,55	6
	Complication risk	13,14	5
	Specificity	17,07	2
	Sensitivity	22,98	1
	Cost	*	*
US	Process	15,65	3
	Frequency	4,85	7
	Follow-up	n.s.	n.s.
	Pain	17,20	2
	Preparation	7,96	6
	Complication risk	14,10	5
	Specificity	15,56	4
	Sensitivity	24,68	1
	Cost	*	*

**Study**	**Attributes**	**RI**	**Score**
Van Dam et al. 2010			
Screening-naive	Pain	6,67	4
	Risk of complications	3,44	6
	Location	n.s.	n.s.
	Preparation	21,08	2
	Duration	5,16	5
	Interval	8,60	3
	Risk reduction of death from CRC	55,05	1
Participants	Pain	5,90	5
	Risk of complications	3,33	6
	Location	0,26	7
	Preparation	22,56	2
	Duration	6,15	4
	Interval	8,46	3
	Risk reduction of death from CRC	53,33	1
Nayaradou et al. 2010**	Screening proposed by	n.s.	n.s.
	Process		4
	Sensitivity		1
	Unnecessary colonoscopy	n.s.	n.s.
	CRC mortality reduction		2
	Results transmitted by	n.s.	n.s.
	Cost		3
Groothuis-Oudshoorn et al. 2014	Preparation	18,75	3
	Technique	27,96	1
	Sensitivity	21,71	2
	Specificity	10,20	5
	Complications	8,88	6
	Frequency	12,50	4
Mansfield et al. 2018<br>Class 1	What can the test find? How often should you get the test?	46,08	1
	Can the test remove cancer and polyps?	4,19	4
	Preparation before the test	2,07	5
	Discomfort and activity limitations during and after the test	5,55	3
	Out-of-pocket cost to you	42,11	2
Class 2	What can the test find? How often should you get the test?	16,38	4
	Can the test remove cancer and polyps?	27,69	2
	Preparation before the test	n.s.	n.s.
	Discomfort and activity limitations during and after the test	19,31	3
	Out-of-pocket cost to you	36,63	1
Class 3	What can the test find? How often should you get the test?	4,43	2
	Can the test remove cancer and polyps?	1,69	4
	Preparation before the test	n.s.	n.s.
	Discomfort and activity limitations during and after the test	2,09	3
	Out-of-pocket cost to you	91,78	1
Osborne et al. 2018	Sample type	13,03	3
	Test performance	67,60	1
	Cost	19,37	2
Bekker-Grob et al. 2019	Effectiveness	4,71	4
	False negative	n.s.	n.s.
	Frequency	38,14	1
	Waiting time diagn test	21,58	3
	Waiting time follow-up test	35,57	2

Study	Attributes	RI	Score
Phisalprapa et al. 2021			
Individuals without symptoms	Pain	n.s.	n.s.
	Risk reduction of CRC-related mortality (%)	45,32	1
	Risk of complications	7,68	4
	Screening interval	11,10	3
	Bowel preparation	6,48	5
	Out-of-pocket cost (Thai Baht, THB)	29,43	2
Discrete Choice Experiments – Labelled – Coefficients			
Hol et al. 2010			
Screening-naive	Risk reduction of CRC-related mortality		
	FOBT	44,83	2
	Sigmoidoscopy	36,31	2
	Colonoscopy	35,71	2
	Screening interval		
	FOBT	55,17	1
	Sigmoidoscopy	63,69	1
	Colonoscopy	64,29	1
Participants	Risk reduction of CRC-related mortality		
	FOBT	49,24	2
	Sigmoidoscopy	53,72	1
	Colonoscopy	42,27	2
	Screening interval		
	FOBT	50,76	1
	Sigmoidoscopy	46,28	2
	Colonoscopy	57,73	1
Benning et al. 2014	Stool		
	Blood		
	Combi		
	Sensitivity stool/blood	58,18	1
	Unnecessary follow-up test stool/blood	n.s.	n.s.
	Unnecessary follow-up test stool/blood	n.s.	n.s.
	Risk reduction stool/blood	26,67	2
	Level of evidence (strong) stool/blood	15,15	3
	Sensitivity combi	41,76	1
	Unnecessary follow-up test combi	n.s.	n.s.
	Unnecessary follow-up test combi	n.s.	n.s.
	Risk reduction combi	40,00	2
	Level of evidence (strong) combi	18,24	3

CRC=colorectal cancer; GP=general practitioner; N/A=no coefficient reported; n.s.=not significant
†Women; ‡Men; *No information on measurement unit of continuous attribute; **Relative importance as reported by authors of included studies
